# *APOE* genotype and biological age impact inter-omic associations related to bioenergetics

**DOI:** 10.18632/aging.206243

**Published:** 2025-05-03

**Authors:** Dylan Ellis, Kengo Watanabe, Tomasz Wilmanski, Michael S. Lustgarten, Andres V. Ardisson Korat, Gwênlyn Glusman, Jennifer Hadlock, Oliver Fiehn, Paola Sebastiani, Nathan D. Price, Leroy Hood, Andrew T. Magis, Simon J. Evans, Lance Pflieger, Jennifer C. Lovejoy, Sean M. Gibbons, Cory C. Funk, Priyanka Baloni, Noa Rappaport

**Affiliations:** 1 Institute for Systems Biology, Seattle, WA 98109, USA; 2 Metabolism and Basic Biology of Aging, Jean Mayer USDA Human Nutrition Research Center on Aging at Tufts University, Boston, MA 02111, USA; 3 Jean Mayer USDA Human Nutrition Research Center on Aging at Tufts University, Boston, MA 02111, USA; 4 Department of Biomedical Informatics and Medical Education, University of Washington, Seattle, WA 98195, USA; 5 West Coast Metabolomics Center, University of California, Davis, CA 95616, USA; 6 Institute for Clinical Research and Health Policy Studies, Tufts Medical Center, Boston, MA 02111, USA; 7 Department of Bioengineering, University of Washington, Seattle, WA 98195, USA; 8 Phenome Health, Seattle, WA 98109, USA; 9 Thorne HealthTech, New York, NY 10019, USA; 10 Department of Immunology, University of Washington, Seattle, WA 98195, USA; 11 Paul G. Allen School of Computer Science and Engineering, University of Washington, Seattle, WA 98195, USA; 12 Buck Institute for Research on Aging, Novato, CA 94945, USA; 13 eScience Institute, University of Washington, Seattle, WA 98195, USA; 14 Department of Genome Sciences, University of Washington, Seattle, WA 98195, USA; 15 Present address: Department of Medical Artificial Intelligence and Data Science, Graduate School of Biomedical Sciences, Tokushima University, Tokushima 770-8503, Japan; 16 Present address: School of Health Sciences, Purdue University, West Lafayette, IN 47907, USA

**Keywords:** apolipoprotein E (APOE), biological age, metabolism, Alzheimer’s disease (AD), insulin resistance

## Abstract

Apolipoprotein E (*APOE*) modifies human aging; specifically, the ε2 and ε4 alleles are among the strongest genetic predictors of longevity and Alzheimer’s disease (AD) risk, respectively. However, detailed mechanisms for their influence on aging remain unclear. In the present study, we analyzed multi-omic association patterns across *APOE* genotypes, sex, and biological age (BA) axes in 2,229 community dwelling individuals. Our analysis, supported by validation in an independent cohort, identified diacylglycerols as the top *APOE*-associated plasma metabolites. However, despite the known opposing aging effects of the allele variants, both ε2- and ε4-carriers showed higher diacylglycerols compared to ε3-homozygotes. ‘Omics association patterns of ε2-carriers and increased biological age were also counter-intuitively similar, displaying significantly increased associations between insulin resistance markers and energy-generating pathway metabolites. These results demonstrate the context-dependence of the influence of *APOE*, with ε2 potentially strengthening insulin resistance-like pathways in the decades prior to imparting its longevity benefits. Additionally, they provide an atlas of *APOE*-related ‘omic associations and support the involvement of bioenergetic pathways in mediating the impact of *APOE* on aging.

## INTRODUCTION

Aging is accompanied by a progressive decrease in physiological integrity, which results from the accumulation of damage in different molecular systems and is characterized by genomic instability, deregulated nutrient-sensing, mitochondrial dysfunction, and cellular senescence [1]. Older age increases the risk of death and is the biggest predictor of neurodegenerative disorders such as Alzheimer’s disease (AD), which is the leading cause of dementia [2]. We and others have shown that aging does not occur at the same rate for each individual, implying that a person’s chronological age (CA) is an imprecise measure of their biological age (BA) [3–5]. The difference (i.e., delta) between BA and CA (BA − CA) can be used to represent an individual’s health state normalized for their CA, with a negative delta signifying better health and a positive delta signifying worse health. In effect, delta age can represent the degree to which an individual is biologically aging at an accelerated vs. decelerated pace as compared to chronologically aging. BA, and therefore delta age, are modifiable through lifestyle choices [3], and are thus of interest for designing, proposing, and evaluating wellness interventions.

The haplotypes of the human apolipoprotein E gene (*APOE*) exert strong, divergent effects on aging, with the ε4 allele being the greatest genetic predictor of late onset AD incidence [6–8], whereas the ε2 allele is protective against AD risk [9, 10], and is a predictor itself of longevity, independent of AD [11, 12]. However, despite *APOE*’s long established connection to AD incidence and longevity, the mechanisms underlying its apparent influence on aging and neurodegeneration remain largely uncharacterized. Recent research trends have supported metabolic and immuno-metabolic hypotheses of AD etiology, pointing to perturbations within mitochondrial function, impairments in glucose metabolism and other bioenergetic alterations both peripherally and within the brain as potentially causal mechanisms for dementia and the associated hallmark accumulation of amyloid beta (Aβ) [13–20]. We and others hypothesize that *APOE* could be partly responsible for the complex, interwoven shifts seen in aging and AD, with *APOE* ε4 influencing both brain and blood metabolomes [16, 21–23]. A recent case report of an individual homozygous for the *APOE* Christchurch mutation, resistant to a familial *PSEN1* mutation, suggests *APOE*’s effects are upstream of amyloid production [24]. This aligns with our recent analysis of the Alzheimer’s Disease Neuroimaging Initiative (ADNI) data showing that *APOE*’s reduced ability to off-load excess cholesterol, as well as the redistribution of cholesterol and other fatty acids across cell types in the brain, disrupts metabolic support for neurons by interfering with G protein-coupled receptor (GPCR) signaling in the astrocyte-neuron lactate shuttle [25]. Understanding how different forms of *APOE* affect health throughout life and the context-dependency of its systemic influences on metabolism and aging could provide targets and help in preventing AD. Given the complexity of multifaceted phenotypes such as aging and AD, it is important to investigate beyond changes in individual measurements, and examine how networks of interacting biological features are altered. We thus set out to further understand the effects of *APOE* on system dynamics.

We studied multi-omic data from an AD-undiagnosed cohort of 2,229 community dwelling individuals aged 19–83, investigating the impact of *APOE* genotype and delta age on inter-omic associations (those spanning different types of molecular phenotypic data, for example between clinical chemistries and the metabolome). Our results indicate that *APOE* ε2 carriers and ε4 carriers display a similar increased abundance of plasma diacylglycerols (DAGs) and modified associations in bioenergetic pathways, including changes in ε2-carrying males resembling those of biologically older males. Our results provide an atlas for intervention targets to potentially reduce AD risk and promote longevity, and further contextualize the complex relationship between *APOE*, biological aging, and insulin resistance.

## RESULTS

### Study design and cohort summary

**Figure 1:**
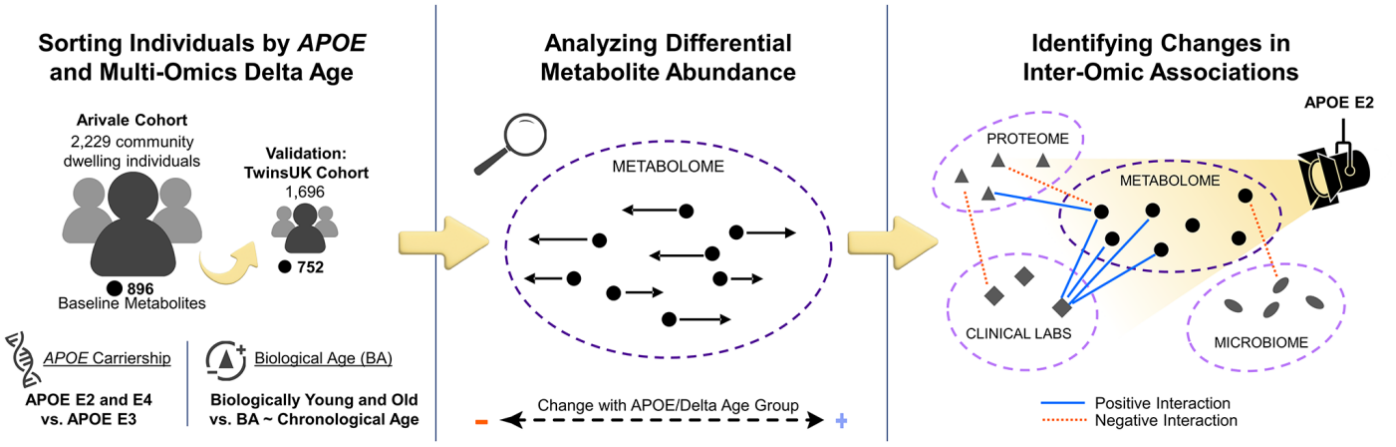
**Study design to identify *APOE* genotype- and delta age-related alterations in the metabolome and inter-omic associations.** Community dwelling individuals from the Arivale cohort were sorted based on delta age and *APOE* ε2 or ε4 carrier status. Metabolomic changes across APOE and delta age statuses were then analyzed. Finally, an inter-omic interaction analysis was performed to identify the effect modification of APOE or delta age status on inter-omic associations. These findings elucidate potential context-dependent relationships within APOE status and delta age group. Analyses were then repeated for validation with the TwinsUK cohort.

This study aimed to analyze how *APOE* genotype and delta age (BA − CA) are associated with shifts in blood metabolomes and inter-omic associations in community dwelling individuals without an AD diagnosis, using data from the Arivale [26] and TwinsUK [27] cohorts (Figure 1). Differential metabolite abundances were first analyzed across *APOE* genotypes and delta age groups. This analysis was followed by an inter-omic interaction analysis, wherein the influence of a condition of interest, such as APOE status (APOE E2 for *APOE* ε2/ε2 or ε2/ε3; APOE E3 for ε3/ε3; or APOE E4 for ε3/ε4 and ε4/ε4) or delta age status, on the association between two analytes of different ’omes was evaluated. The significant inter-omic interactions observed with each sex-stratified APOE status and delta age status were compared.

Baseline data from the Arivale Scientific Wellness dataset [26] was used as a discovery cohort. The Arivale cohort, a former consumer-facing wellness company, consists of subscribers who were deeply-phenotyped and provided with personalized health coaching. Participants ranged from 19 to 83 (mean 46.6) years of age and represented the health of the communities they were drawn from [26] (Supplementary Figure 1). Multi-omic BAs were previously calculated [3], and delta age statuses were defined as biologically older for those with BA at least 7.5 years (~one standard deviation) older than CA, and biologically younger for those with BA at least 7.5 years younger than CA for males and females. Delta age status categorization serves to identify differences in phenotypic measures across individuals aging biologically at a rate slower than, on pace with, or faster than their CA, independent of base CA. We did not observe differences in delta age across APOE status (Supplementary Figure 2). We used data from TwinsUK as a validation cohort, which was originally intended to investigate rheumatologic diseases in identical twins in the United Kingdom, and has since expanded to encompass over 15,000 volunteer identical and non-identical twins [27]. The subsection of the cohort with plasma metabolomics [28] and clinical lab data available for use in this study was 96.4% female, 99.9% non-Hispanic White, and had an older population than Arivale with ages ranging 32 to 87 (mean 58.1) years at baseline. Table 1 provides a demographic summary of the Arivale and TwinsUK cohorts. Using the same method as previously performed on Arivale data, a metabolomics-based BA was calculated for TwinsUK individuals in this study (Pearson’s r = 0.778 for females, 0.776 for males, see Methods and Supplementary Figure 3), with the delta age status cutoff defined as 7.5 years for females and 5.0 years for males, reflective of their standard deviations. In TwinsUK, delta age was found to be increased in APOE E4 in females but decreased in APOE E4 in males (Supplementary Figure 4).

**Table 1:** Summary of Arivale and TwinsUK cohorts.

	**Arivale**		**TwinsUK**	
	**Females**	**Males**	**Females**	**Males**
***N***	1403	826	1635	61
**Age (years)**	46.6 (21.0–83.0)	44.5 (19.0–80.0)	51.4 (32.9–73.7)	51.1 (33.6–58.5)
**Race/Ethnicity (% non-Hispanic whites)**	74.4%	66.0%	99.9%	98.4%
**BMI (kg/m2)**	28.6 (16.9–62.3)	27.7 (18.0–61.5)	25.4 (16.5–48.2)	26.6 (20.3–36.3)
**% Cholesterol medication use**	9.1%	14.0%	3.2%	3.3%
**% APOE E2**	11.3%	10.2%	13.1%	9.8%
**% APOE E3**	63.6%	65.3%	63.1%	52.5%
**% APOE E4**	22.7%	23.2%	21.3%	37.7%
**Delta Age (years)**	0.5 (−28.0–+41.2)	−0.4 (−35.0–43.1)	−2.6 (−25.4–+22.4)	1.8 (−8.3–+16.7)

Continuous data (age, BMI, delta age) are reported as ‘mean (range)’. All other fields are reported as percentage, with E2 representing ε2/ε2 and ε2/ε3, E3 representing ε3/ε3, and E4 representing ε3/ε4 and ε4/ε4. Data reported for each cohort is for individuals directly prior to use for interaction analysis. Baseline data was used for both Arivale and TwinsUK. Delta age data of the Arivale cohort was derived from the combined BA model predictions.

### Diacylglycerols and plasmalogens are the metabolites most significantly associated with *APOE* genotype and delta age status

**Figure 2:**
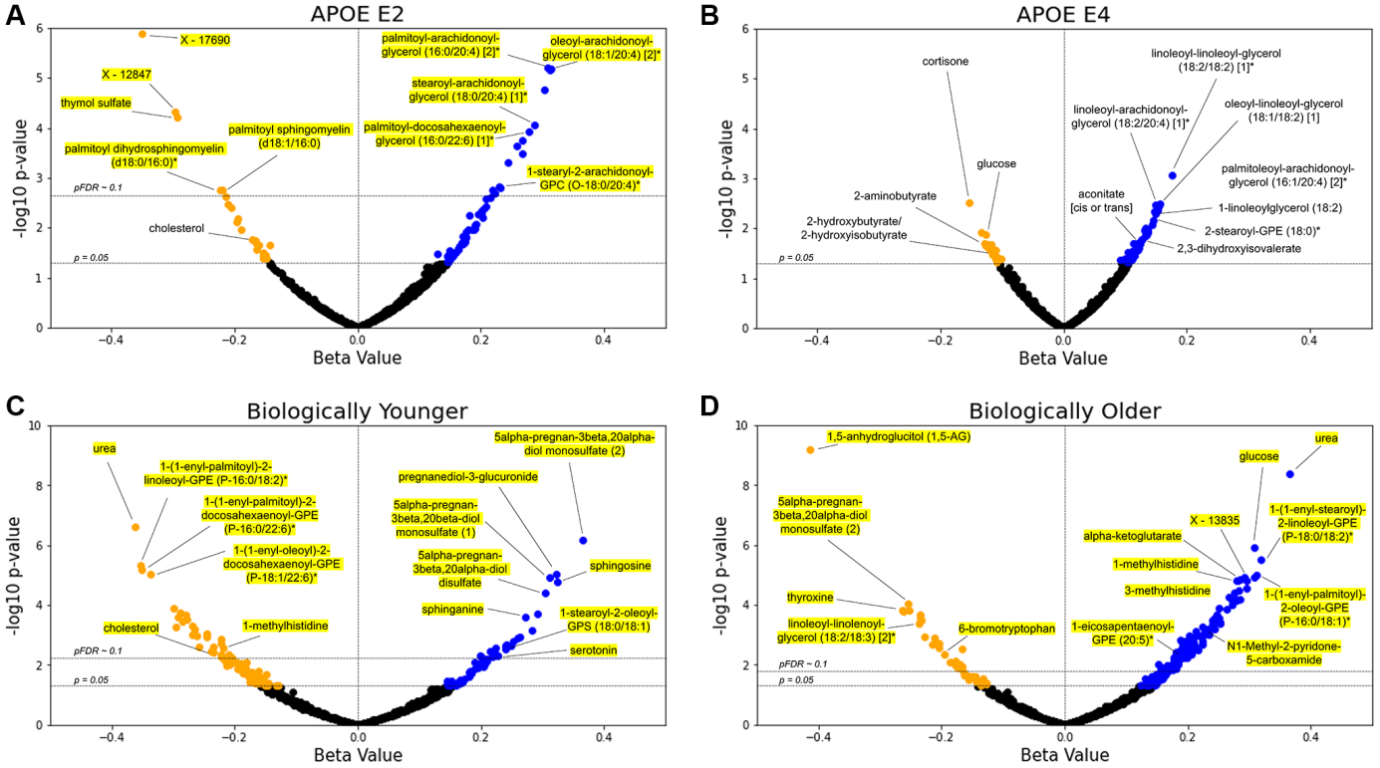
**Lipids are the main APOE- and delta age-associated metabolites.** (**A**–**D**) Volcano plots for the APOE E2 (**A**), APOE E4 (**B**), biologically younger (**C**), or biologically older (**D**) groups. For each metabolite, presented are the *β*-coefficient estimate and its log10 *p*-value from the GLM including metabolite abundance as the dependent variable, group statuses as the independent variables, and age, BMI, use of cholesterol medications, sex, and first two genetics principal components as the covariates (see Methods). Blue data points indicate a positive association between metabolite and test group with pre-adjusted *p* < 0.05, whereas orange points indicate a negative pre-adjusted association. Yellow highlighting indicates significance after multiple hypothesis testing (pFDR < 0.1, Benjamini–Hochberg method). *n* = 896 metabolites.

To analyze the associations of *APOE* and delta age group with the metabolome, we constructed two sets of generalized linear models (GLMs) for each metabolite: one using metabolite abundance as the dependent variable (log2 transformed, then z-scored for comparability of *β*-coefficient estimates), APOE E2 and E4 statuses as the independent variables, and covariates (age, body mass index, use of cholesterol medications, sex, and first two genetics principal components) and the other using biologically younger and older delta age groups in place of APOE status (see Methods). Figure 2 depicts the distribution of *β*-coefficient estimates and their significance for the experimental variables from these models. Out of 896 metabolites, 87 differential metabolite abundance GLMs had APOE E2 *β*-coefficient estimates with pre-adjusted *p* < 0.05, while 67 had pre-adjusted *p* < 0.05 for E4. After adjusting for false discovery rate (FDR, Benjamini-Hochberg method with 5% FDR used throughout), 20 metabolites retained significant associations at pFDR < 0.1 for APOE E2 (Figure 2A, 2B). Top positively APOE E2-associated metabolites included DAGs such as linoleoyl-arachidonoyl-glycerol (18:2/20:4) [1]* (*β*-coefficient estimate = 0.312), palmitoyl-arachidonoyl-glycerol (16:0/20:4) [2]* (*β* = 0.309), and oleoyl-arachidonoyl-glycerol (18:1/20:4) [2]* (*β* = 0.315) (all pFDR = 1.58e-3), and top negatively APOE E2-associated metabolites included sphingolipids such as palmitoyl dihydrosphingomyelin (d18:0/16:0)* (*β* = −0.224) and palmitoyl sphingomyelin (d18:1/16:0) (*β* = −0.220) (both pFDR = 0.089). Thymol sulfate was another top negatively E2-associated metabolite (*β* = −0.292, pFDR = 7.72e-3). For APOE E4, DAGs trended toward positive associations though were not significant after FDR-adjustment, including linoleoyl-linoleoyl-glycerol (18:2/18:2) [1]* (*β* = 0.177, pre-adjusted *p* = 8.58e-4), oleoyl-linoleoyl-glycerol (18:1/18:2) [1] (*β* = 0.156, *p* = 3.22e-3) and linoleoyl-arachidonoyl-glycerol (18:2/20:4) [1]* (*β* = 0.150, *p* = 3.45e-3) (all had pFDR = 0.514). Of the 13 DAGs included in the Arivale dataset, all were positively associated pre-adjustment (*p* < 0.05) with E2 (8 out of 13 also with pFDR < 0.1, post-adjustment), while 4 were positively associated pre-adjustment with E4. Those DAGs associated with E4 pre-adjustment mostly contained linoleoyl acyl groups, while those with stronger significant associations with E2 tended to contain more palmitoyl, oleoyl, and stearoyl groups. We further performed enrichment analysis for the metabolites significantly (pFDR < 0.1) positively or negatively associated with APOE E2 and E4 using the sub-pathways annotated by the Metabolon platform (Supplementary Table 1). DAGs were enriched in the positive APOE-associated metabolites for the E2 group (pFDR = 2.55e-12). We also performed enrichment analysis on those metabolites with pre-adjusted (*p* < 0.05) positive and negative associations with APOE E2 and E4 (Supplementary Table 2). In addition to the enrichment of DAGs, these sets of associations with pre-adjusted *p* < 0.05 were enriched for plasmalogens and long chain fatty acids in the positive associations with APOE E2 (pFDR = 0.075 for both). The sphingolipid metabolism sub-pathway was enriched in negative APOE E2 associations (pFDR = 2.33e-5). For the positive associations with APOE E4, lysolipids were enriched (pFDR = 0.091). DAGs were also narrowly outside FDR significance (pFDR = 0.111, *p* = 2.61e-3) for positive associations in APOE E4, showing a similar trend to APOE E2 despite the expected opposite effect of these genotypes.

For the delta age analyses using GLMs (Figure 2C, 2D), 51 metabolites for the biologically young group were significant at pFDR < 0.1 (158 metabolites had pre-adjusted *p* < 0.05), while 143 were significantly associated with the biologically old group (227 were associated pre-adjustment). Urea was the top metabolite negatively associated with the biologically young group (*β* = −0.362, pFDR = 2.22e-4) and the second top metabolite positively associated with the biologically old group (*β* = 0.366, pFDR = 1.80e-6). For the biologically young, we observed positive associations for steroid metabolites such as 5alpha-pregnan-3beta, 20alpha-diol monosulfate (2) (*β* = 0.365, pFDR = 2.89e-4) and pregnanediol-3-glucuronide (*β* = 0.323, pFDR = 1.38e-3) among the most significant, as well as sphingosine (*β* = 0.325, pFDR = 1.86e-3) and sphinganine (*β* = 0.273, pFDR = 0.016). For the biologically old, 1,5-anhydroglucitol (1,5-AG), a known marker for glycemic control that is inversely associated with diabetes risk [29, 30], had the most significant *β*-coefficient estimate and was negatively associated (*β* = −0.414, pFDR = 5.64e-7). Glucose (*β* = 0.308, pFDR = 3.65e-4) and alpha-ketoglutarate (*β* = 0.281, pFDR = 1.30e-3), central bioenergetic metabolites, were the third and tenth most significant metabolites for the biologically old, both positively associated, while neither had FDR significant associations for the biologically young. 1-methylhistidine was also near the top of the list of significance for both groups, being negatively associated with the biologically young (*β* = −0.219, pFDR = 0.068) but positively associated with the biologically old (*β* = 0.279, pFDR = 1.30e-3). The following enrichment analysis on the significant (pFDR < 0.1) associations with delta age statuses (Supplementary Table 1) revealed that plasmalogens, phospholipids crucial for cell membrane integrity and linked to important roles in cognitive health and neurological function, were the most significantly enriched sub-pathway in metabolites negatively associated with the biologically young (pFDR = 4.28e-5) and in metabolites positively associated with the biologically old (pFDR = 8.52e-8). Steroid metabolites were also enriched in positive associations with the biologically young (pFDR = 0.088), and the polyamine metabolism subpathway was enriched in the negative associations with the biologically old (pFDR = 0.030).

Between the biologically young and old, 21 metabolites were FDR-significant for both groups, all having diverging associations. Comparing the analyses of APOE and delta age status, two of the metabolites significantly (pFDR < 0.1) associated with APOE E2 were also significantly (pFDR < 0.1) associated with the biological old status: linoleoyl-linoleoyl-glycerol (18:2/18:2) [1]* was positively associated with APOE E2 but negatively associated with the biological old, while palmitoyl dihydrosphingomyelin (d18:0/16:0)* was negatively associated with APOE E2 but positively associated with the biological old. Several metabolites have associations with pre-adjusted *p* < 0.05 overlapping across experimental groups: 13 metabolites are associated concordantly with APOE E2 and biologically younger individuals, while 4 metabolites are associated discordantly. Between APOE E2 and biologically older individuals, 12 metabolites have concordant associations while 8 are discordant. For APOE E4 and biologically younger individuals, 1 metabolite is concordantly associated while 5 are discordantly associated. Finally, between APOE E4 and biologically older individuals, 14 metabolites have concordant associations and 7 have discordant associations. In a sex-stratified analysis, biologically old males and females also shared a significant (pFDR < 0.1) negative association with 1,5-AG and significant (pFDR < 0.1) positive associations with urea, glucose, and 1-palmitoyl-2-oleoyl-GPC (16:0/18:1) (Supplementary File 1).

An additional set of stratified analyses was performed with the same models for the subsets of the Arivale cohort in the bottom, middle, and top tertiles of CA to examine which metabolites associate with delta age and APOE statuses at different stages of life (Supplementary Table 3). Sphingosine was the only metabolite with pFDR < 0.1 in any association with biological young, being positively associated in the bottom tertile (*β* = 0.582, pFDR = 0.081). Many of the 143 metabolites significantly associated with the biologically old status in the non-stratified analysis retained significant associations within the CA tertiles (12, 43, and 18 for the bottom, middle, and top tertiles), whereas a number of formerly non-significant metabolites reached statistical significance (4, 55, and 13 for the bottom, middle, and top tertiles). Of those significantly associated, two metabolites carried discordant associations across tertiles, with 1-linolenoyl-GPC (18:3)* and indoleacetate being positively associated with the biologically old status in the middle tertile (*β* = 0.375 and 0.369, both pFDR = 0.062) but negatively associated in the top tertile (*β* = −0.350 and −0.351, both pFDR = 0.080). No metabolite associated with the biologically old status within any of the CA tertiles with pre-adjusted *p* < 0.05 carried the opposite signed association with pre-adjusted *p* < 0.05 in the non-stratified analysis. For APOE, the DAGs linoleoyl-arachidonoyl-glycerol (18:2/20:4) [1]* and palmitoleoyl-arachidonoyl-glycerol (16:1/20:4) [2]* were positively associated with APOE E4 in the bottom CA tertile (*β* = 0.357 and 0.346, both pFDR = 0.047) whereas DAGs oleoyl-arachidonoyl-glycerol (18:1/20:4) [2]*, stearoyl-arachidonoyl-glycerol (18:0/20:4) [2]*, and stearoyl-arachidonoyl-glycerol (18:0/20:4) [1]* were positively associated with APOE E2 in the top CA tertile (*β* = 0.453, 0.454, and 0.451; all pFDR = 0.070) (Supplementary File 2).

### Bioenergetic analyte associations are modified by APOE and delta age status

To explore systemic and context-dependent omics changes associated with *APOE* and delta age, we assessed the associations between 509,360 inter-omic pairs (being of different ‘omes) of analytes across the plasma metabolome, plasma proteome, gut microbiome, and clinical chemistries using an analyte-by-experimental group (i.e., APOE E2, APOE E4, biologically young, or biologically old) interaction term in the GLMs for each pair (see Methods). This type of statistical test, called an interaction analysis, assesses whether the relationship between two analytes is dependent on a third variable (in this case, the experimental group). The association between two analytes can be positively or negatively modified by a third variable, such as the association between glucose and *Klebsiella* being more positive in APOE E2 than in E3 in males in this study. Other recent works have successfully employed interaction analyses to identify multi-omic differences in COVID-19 disease states [31] and to examine how the associations between proteins and AD incidence are modified by *APOE* ε4 carriership [32], as examples.

The ten analyte pairs with the lowest *p*-values for the interaction term in each sex-stratified experimental group model are presented in Table 2, and the top ten pairs for models using allele dosage and continuous delta age are presented in Supplementary Table 4. Individual associations, or lack thereof, between analytes and either APOE or delta age did not appear to influence whether analytes were identified in the interaction analysis, suggesting that the interaction analysis provides a unique layer of information. Indeed, out of the 79 metabolites appearing in the top twenty significant analyte pairs of all the APOE-related interaction analyses, 65 were detected as significant exclusively in the interaction analysis and not (*p* > 0.05) in the earlier differential metabolite abundance analysis, including fumarate, malate, and ribitol. Similarly, for the delta age-related interaction analyses, 37 out of the 56 metabolites in the top twenty pairs of each were exclusively significant in the interaction analyses, including pyruvate, lactate, and glutamate. For APOE E2 males, 60 significant (pFDR < 0.1) interactions were identified, including positively modified associations between glucose and *Klebsiella*; triglycerides and ribitol; as well as both glucose and phenol sulfate. *APOE* ε2 allele dosage significantly modified 17 inter-omic associations, including positively modified bioenergetic associations such as HbA1c with malate and fumarate as well as glucose with aconitate (cis or trans), as well as negatively modified associations between both LDL particle number and LDL small particle number with LDLR. *APOE* ε4 allele dosage significantly modified 5 inter-omic associations, including negatively modifying the association between isoursodeoxycholate and both *Rikenellaceae RC9 gut group* and *Prevotellaceae UCG-001*. Biologically younger males and females exhibited 28 and 16 significant interaction pairs, respectively, with SRC (proto-oncogene non-receptor tyrosine kinase Src protein) appearing frequently in negatively modified associations with several metabolites in the males and IL6 appearing in positively modified associations with N-acetylglutamine but negatively modified associations with *Agathobacter*. Biologically older males and females significantly modified 526 and 89 associations, respectively, including many positively modified associations containing glucose or HbA1c from the clinical chemistries with bioenergetic metabolites such as pyruvate and alpha-ketoglutarate. Continuous delta age showed a similar signature in its 840 significantly modified associations, including positively modifying the association between HbA1c and pyruvate, mannose, lactate, CD163, gluconate, and fructose.

**Table 2:** Top ten inter-omic analyte pair associations modified by each APOE and delta age status, stratified by sex.

**APOE Status**											
**Female APOE E2**			**Female APOE E4**			**Male APOE E2**			**Male APOE E4**		
++	BMP6	N-palmitoylglycine	−−	isoursodeoxycholate	Rikenellaceae_RC9_gut_group	++	homocitrulline	Megasphaera	−−	GDF15	X - 15461
−	AMBP	eicosapentaenoate (EPA; 20:5n3)	−−	isoursodeoxycholate	Prevotella_2	++	TGFA	Megamonas	+	Total Bilirubin	C-glycosyltryptophan
−	SULT1A1	phosphoethanolamine	−	isoursodeoxycholate	Anaeroplasma	++	TGFA	Megasphaera	+	LDL Size	1-palmityl-2-oleoyl-GPC (O-16:0/18:1)*
−	phenylpyruvate	GCA-900066225	+	ITGB2	X - 21258	++	glucose	Klebsiella	+	High-sensitivity CRP	taurocholate
−	IL18	cysteine s-sulfate	+	Lymphocytes	lactate	++	hydroxyasparagine**	DTU089	+	LDL Size	1-(1-enyl-oleoyl)-2-docosahexaenoyl-GPE (P-18:1/22:6)*
+	BMP6	2-arachidonoyl-GPC (20:4)*	+	1-arachidonoyl-GPE (20:4n6)*	Tyzzerella	++	epiandrosterone sulfate	Lachnospiraceae_UCG-008	−	Arachidonic acid	1-adrenoyl-GPC (22:4)*
+	FGF23	eicosanedioate (C20-DC)	+	CD6	1-palmitoyl-2-oleoyl-GPE (16:0/18:1)	++	glucose	Enterobacter	+	CCL19	Oxalobacter
−	N-acetylglutamate	Faecalibacterium	−	Triglycerides	X - 18899	++	Alkaline Phosphatase	CHI3L1	+	X - 15461	Allisonella
+	FGF23	2-aminooctanoate	−	IL7	X - 18901	++	hydroxyasparagine**	Megasphaera	+	TFF3	3-methylglutarylcarnitine (2)
−	CD93	X - 17676	−	ursodeoxycholate	Rikenellaceae_RC9_gut_group	++	CD4	1-eicosapentaenoyl-GPE (20:5)*	+	mannitol/sorbitol	Turicibacter
**Delta age status**											
**Female Bio. Younger**			**Female Bio. Older**			**Male Bio. Younger**			**Male Bio. Older**		
++	X - 12216	Faecalibacterium	++	ACE2	alpha-ketoglutarate	++	CCL19	mannitol/sorbitol	−−	CHIT1	X - 12544
−−	cinnamoylglycine	Anaeroplasma	++	Hemoglobin A1C	alpha-ketobutyrate	++	CD4	X - 15461	++	Red Blood Cell Distribution Width	taurochenodeoxycholate
−−	cinnamoylglycine	Prevotellaceae_UCG-001	−−	Urea	UBA1819	−−	SRC	phosphatidylcholine (14:0/14:0, 16:0/12:0)	++	Glucose	N-acetylvaline
++	IL6	N-acetylglutamine	++	ACE2	xanthine	−−	BOC	Ectoine	++	Red Blood Cell Distribution Width	X - 12007
++	IL6	N-acetylglutamine	−−	Bun/Creatinine Ratio	UBA1819	++	hippurate	Alistipes	++	Glucose	linoleoyl-linoleoyl-glycerol (18:2/18:2) [1]*
++	isoursodeoxycholate	Prevotella_2	++	N-stearoyl-sphingosine (d18:1/18:0)*	Enterorhabdus	−−	STK4	phosphatidylcholine (14:0/14:0, 16:0/12:0)	++	Glucose	mannose
++	CCL2	N-acetylvaline	++	Hemoglobin A1C	gluconate	++	Red Blood Cell Distribution Width	isobutyrylcarnitine (C4)	++	Red Blood Cell Distribution Width	taurocholate
−−	IL6	Agathobacter	−−	alpha-hydroxyisocaproate	Faecalibacterium	−−	MMP7	X - 11632	−−	HAVCR1	1-ribosyl-imidazoleacetate*
++	IL6	N-acetyl-1-methylhistidine*	−−	LPL	sphingomyelin (d18:0/20:0, d16:0/22:0)*	−−	CTSL	X - 13835	−−	Mean Corpuscular Hemoglobin	CST5
++	CCL3	6-bromotryptophan	−−	LPL	behenoyl dihydrosphingomyelin (d18:0/22:0)*	−−	SPON2	Ectoine	−−	CHI3L1	behenoyl sphingomyelin (d18:1/22:0)*

For each test group, the ten analyte pairs with the lowest *p*-values for the interaction term representing the modification of APOE or delta age status on the association between the two analytes are tabulated. ‘+’ and ‘−’ indicate positive and negative interaction terms, respectively, with ‘++’ or ‘--’ indicating significance at pFDR < 0.1 (Supplementary File 3 for full data). Underlining indicates a metabolite associated with the experimental group in the analysis of differential metabolite abundance (with pre-adjusted *p* < 0.05). Metabolite names ending in “*” indicate compounds not confirmed based on a standard but having high confidence in its identity, while those ending in “**” indicate compounds for which a standard is not available, but for which there is reasonable confidence in its identity or the information provided.

### Comparison of inter-omic signatures of APOE and delta age status

**Figure 3:**
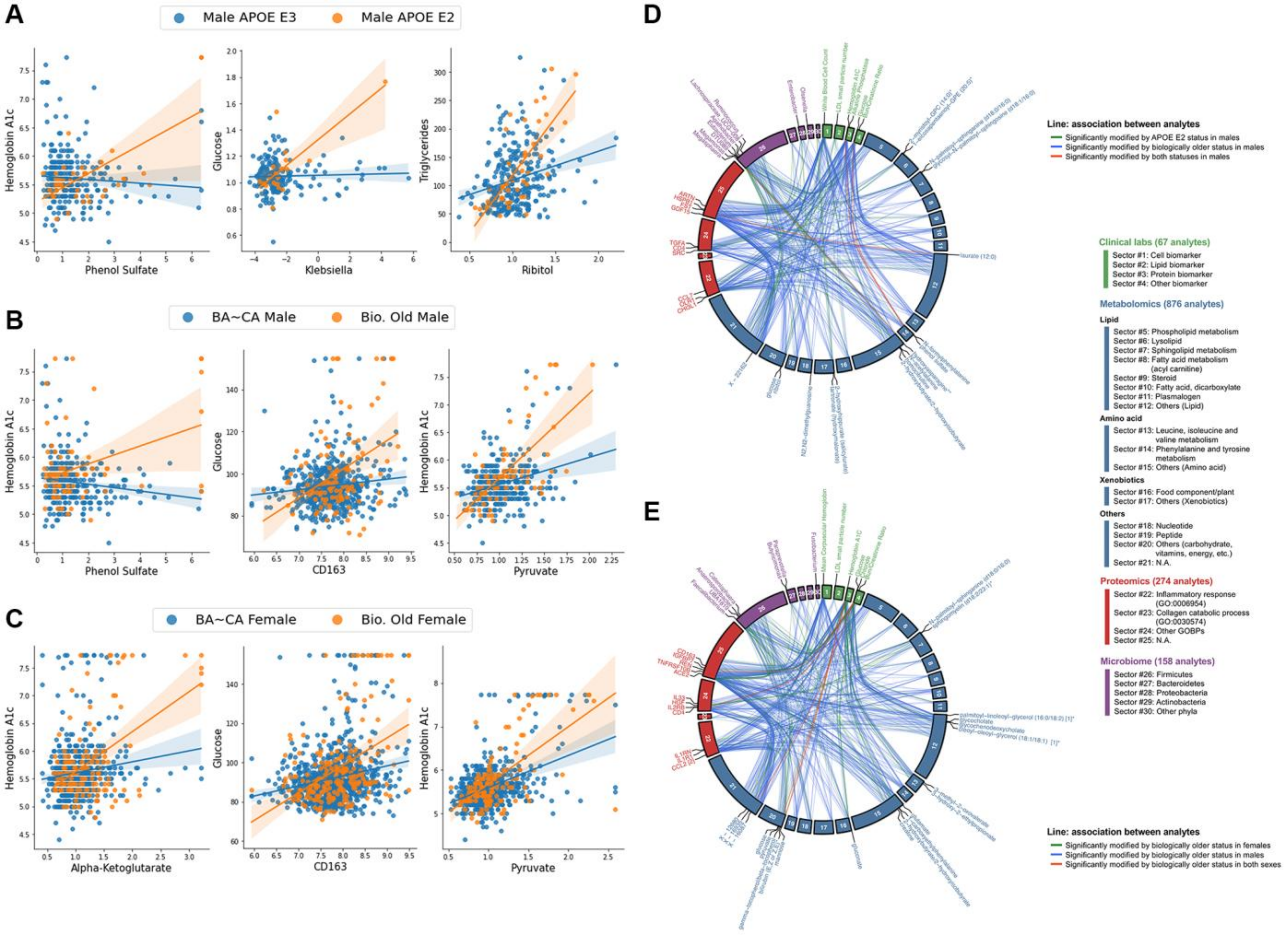
**Biologically older males show similar multi-omic association signatures to APOE E2 males and biologically older females, particularly within central bioenergetic analytes.** (**A**–**C**) Scatter plots of inter-omic analyte pairs with associations significantly modified by APOE E2 in males (**A**), and by biological oldness in males (**B**) and females (**C**). Line indicates simple linear regression, with shading indicating the 95% confidence interval. (**D**, **E**) Circos plots depicting the shared analyte associations (pFDR < 0.1, Benjamini-Hochberg method) between male APOE E2 and biologically older males (**D**), and between biologically older males and females (**E**). Associations specific to one group are connected with green and blue lines, whereas significant concordant associations shared in both groups are presented in red lines. Analyte nodes in associations significant to both groups are labeled.

Analyte pairs having interaction terms with pFDR < 0.1 for each subgroup for both sets of interaction analyses were compared to identify similarities and differences between the contextual manifestation of inter-omic associations of APOE and delta age groups in both males and females. We directly compared the inter-omic association signatures between these groups to characterize the perturbations in these complex systems and to capture the essence of age-related metabolic shifts by pinpointing the changes in associations that converge or diverge across conditions. Figure 3 highlights key modified inter-omic associations and compares the association signatures across APOE E2 males and biologically older males as well as across biologically older males and females. APOE E2 males and biologically older males shared four significant interactions, all being positively modified in both groups: those between hydroxyasparagine** and *Megasphaera*; FST (follistatin protein) and laureate (12:0); HbA1c and phenol sulfate; and glucose and phenol sulfate. Biologically older males and females shared five pairs, with all the associations being positively modified in both: HbA1c and pyruvate; HbA1c and mannose; glucose and HGF (hepatocyte growth factor); glucose and CD163; and glucose and X - 16087 (unknown metabolite). In addition to these modified associations directly shared between biologically old males and females, each group had associations with metabolites from similar pathways modified. For instance, biologically older males showed positively modified associations between HbA1c and aspartate, glutamate, lactate, mannose, and laureate (12:0), while biologically older females had positively modified associations between HbA1c and alpha-ketoglutarate, margarate (17:0), and taurine. The only other significantly modified association overlapping in multiple of these groups was that between isoursodeoxycholate and *Prevotella 2*, which was positively modified in biologically younger females but negatively modified in APOE E4 females. Finally, for the models testing *APOE* allele dosage and continuous delta age, both *APOE* ε2 allele dosage and delta age positively modified the associations between glucose and aconitate (cis or trans), and glucose and 3-hydroxy-2-ethylpropionate (Supplementary Figure 5). More comprehensive results of the inter-omic interaction analyses are included in Supplementary File 3.

### Validation analyses in TwinsUK

**Figure 4:**
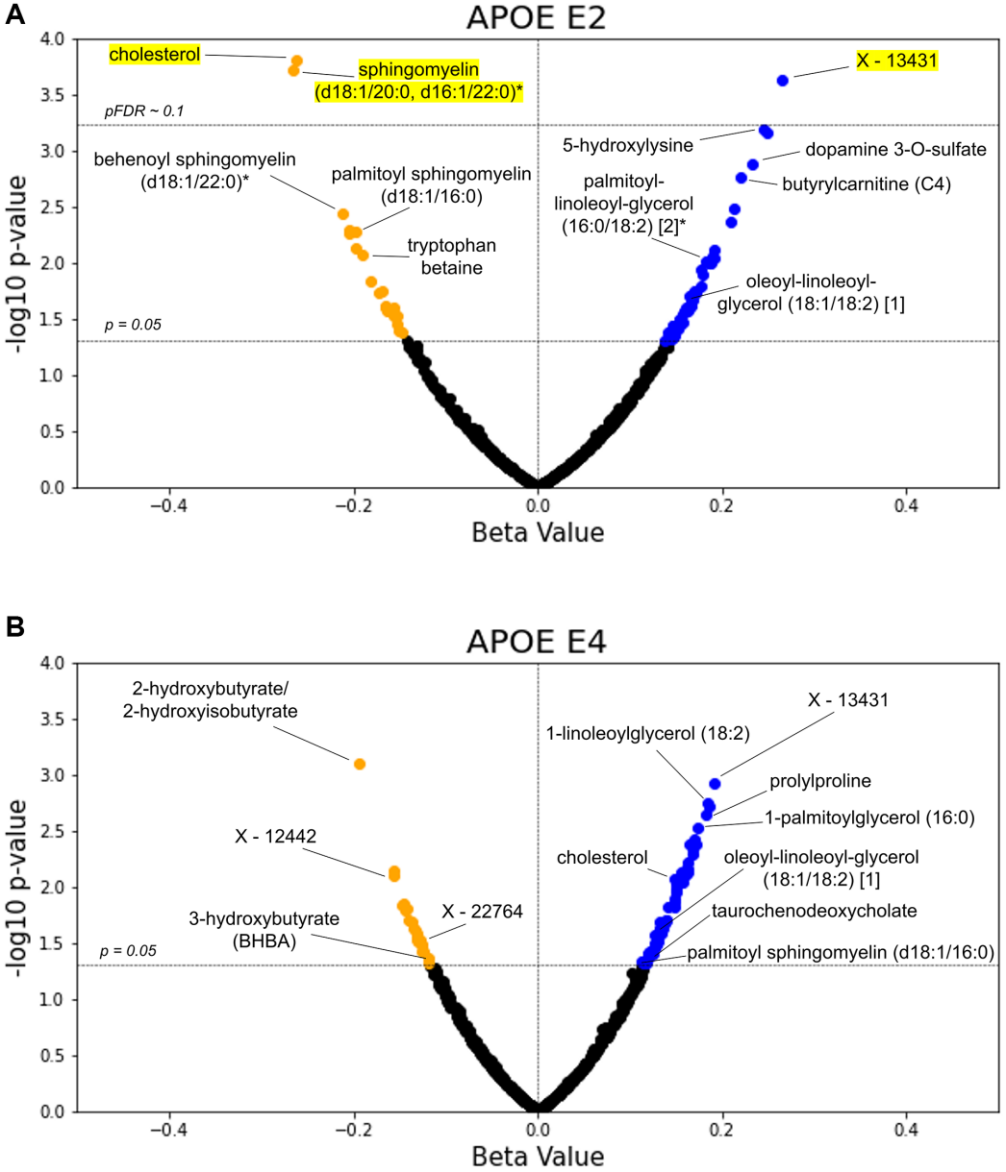
**TwinsUK validates lipids as top APOE associated metabolites.** The *β*-coefficient estimates for the APOE E2 (**A**) and E4 (**B**) groups are plotted against their -log10 pre-adjusted *p*-value from the metabolite GLMs. Blue data points indicate a positive association between metabolite and test group with pre-adjusted *p* < 0.05, whereas orange points indicate a negative pre-adjusted association. Yellow highlighting indicates significance after multiple hypothesis testing (pFDR < 0.1, Benjamini-Hochberg method).

In the individual GLMs (Supplementary File 4), out of 752 total metabolites (with 547 overlapping those in Arivale), 74 associations with APOE E2 and 80 with APOE E4 had *p* < 0.05. After FDR adjustment, three metabolites had pFDR < 0.1 for APOE E2: cholesterol (*β* = −0.262) and sphingomyelin (d18:1/20:0, d16:1/22:0)* (*β* = −0.266) were decreased, and an unidentified metabolite was increased (*β* = 0.265) (all pFDR = 0.059) (Figure 4). Consistent with the Arivale finding, lipids appeared amongst the most significant *APOE*-associated metabolites, and DAGs were significantly enriched in the metabolites with *p* < 0.05 for associations with APOE E2 and E4 (pFDR = 1.10e-3 and 0.036, respectively) (Supplementary Table 2). Analytes involved in sphingolipid metabolism were also significantly enriched in the metabolites with *p* < 0.05 negatively associated with APOE E2 (pFDR = 2.95e-4), as in Arivale (pFDR = 2.33e-4). In stratifying the analysis by CA tertiles, five monoacylglycerols were positively associated with APOE E4 in the bottom tertile; an unidentified metabolite and butyrylcarnitine (C4) were positively associated with APOE E2 in the top tertile; and 1-oleoylglycerol (18:1) and an unidentified metabolite were positively associated with APOE E4, while N-acetylcitrulline was negatively associated in the top tertile (Supplementary Table 3).

We further explored direct comparisons of the metabolites with pre-adjusted *p* < 0.05 in both the Arivale and TwinsUK cohorts (Table 3). Out of the 547 overlapping metabolites tested in Arivale and TwinsUK, nine metabolites had *p* < 0.05 in both cohorts with the same direction for APOE E2: four DAGs, cholesterol, two sphingomyelins, and two other lipids. One metabolite, 4-methylcatechol sulfate, had *p* < 0.05 in both cohorts but in the opposite direction for APOE E2. For APOE E4, 11 metabolites had *p* < 0.05 in both cohorts with the same directional difference, including three DAGs, two monoacylglycerols, and 2-hydroxybutyrate/2-hydroxyisobutyrate. The metabolite associations with delta age groups were not tested in the TwinsUK cohort, because the BA model was able to be generated only from the metabolomics data.

**Table 3:** Comparison of significant metabolites in differential abundance GLM tests for APOE across Arivale and TwinsUK.

**Metabolite**	**Arivale E2**	**Arivale E4**	**TwinsUK E2**	**TwinsUK E4**
1-(1-enyl-stearoyl)-2-arachidonoyl-GPC (P-18:0/20:4)	0.170	–0.116	ns	ns
1-(1-enyl-stearoyl)-2-dihomo-linolenoyl-GPE (P-18:0/20:3)*	0.190	−0.126	Not in Dataset	Not in Dataset
1-(1-enyl-stearoyl)-2-oleoyl-GPE (P-18:0/18:1)	0.163	ns	0.210	ns
1-docosapentaenoyl-GPC (22:5n3)*	ns	0.108	ns	0.148
1-linoleoylglycerol (18:2)	**0.230**	0.150	ns	0.120
1-oleoylglycerol (18:1)	0.202	0.112	ns	0.150
1-stearoyl-2-arachidonoyl-GPE (18:0/20:4)	0.166	ns	0.156	ns
1-stearoyl-2-linoleoyl-GPE (18:0/18:2)*	ns	0.134	ns	0.128
1-stearoyl-GPE (18:0)	ns	0.137	ns	0.160
1-stearyl-2-arachidonoyl-GPC (O-18:0/20:4)*	**0.232**	−0.110	Not in Dataset	Not in Dataset
2-hydroxybutyrate/2-hydroxyisobutyrate	ns	−0.118	ns	−0.194
2-linoleoyl-GPE (18:2)*	ns	0.110	ns	0.148
2-stearoyl-GPE (18:0)*	ns	0.146	ns	0.134
3-(3-hydroxyphenyl)propionate	Not in Dataset	Not in Dataset	−0.166	−0.126
4-methylcatechol sulfate	−0.197	ns	0.149	ns
cholesterol	−0.172	ns	**−0.262**	0.149
diacylglycerol (16:1/18:2 [2], 16:0/18:3 [1])*	0.209	0.112	Not in Dataset	Not in Dataset
isoleucylvaline	Not in Dataset	Not in Dataset	0.165	0.130
linoleoyl-arachidonoyl-glycerol (18:2/20:4) [1]*	**0.312**	0.150	Not in Dataset	Not in Dataset
linoleoyl-linoleoyl-glycerol (18:2/18:2) [1]*	**0.221**	0.177	Not in Dataset	Not in Dataset
oleoyl-arachidonoyl-glycerol (18:1/20:4) [1]*	**0.304**	0.148	Not in Dataset	Not in Dataset
oleoyl-arachidonoyl-glycerol (18:1/20:4) [2]*	**0.315**	0.133	Not in Dataset	Not in Dataset
oleoyl-linoleoyl-glycerol (18:1/18:2) [1]	0.208	0.156	0.166	0.132
oleoyl-linoleoyl-glycerol (18:1/18:2) [2]	0.206	0.127	0.158	0.133
palmitoleoyl-arachidonoyl-glycerol (16:1/20:4) [2]*	**0.222**	0.155	Not in Dataset	Not in Dataset
palmitoleoyl-linoleoyl-glycerol (16:1/18:2) [1]*	0.176	0.132	Not in Dataset	Not in Dataset
palmitoyl dihydrosphingomyelin (d18:0/16:0)*	**−0.224**	ns	−0.157	ns
palmitoyl sphingomyelin (d18:1/16:0)	**−0.220**	ns	−0.198	0.115
palmitoyl-arachidonoyl-glycerol (16:0/20:4) [2]*	**0.309**	0.101	Not in Dataset	Not in Dataset
palmitoyl-linoleoyl-glycerol (16:0/18:2) [1]*	0.197	0.107	0.147	0.119
palmitoyl-linoleoyl-glycerol (16:0/18:2) [2]*	**0.220**	ns	0.182	ns
pyrraline	Not in Dataset	Not in Dataset	0.172	0.123
X - 11491	Not in Dataset	Not in Dataset	0.156	0.122
X - 11795	ns	0.125	ns	−0.137
X - 13431	ns	ns	**0.265**	0.191
X - 24065	Not in Dataset	Not in Dataset	−0.204	0.168

Metabolites with pre-adjusted *p* < 0.05 for at least two out of four APOE *β*-coefficients in metabolite abundance GLMs across tests for Arivale and TwinsUK are reported. β-coefficient estimates are reported for metabolites with pre-adjusted *p* < 0.05, with bolded β-coefficients denoting pFDR < 0.1. Nonsignificance is indicated with ‘ns’ while metabolites missing from one of the two datasets are denoted. Metabolites are sorted alphabetically. Metabolite names ending in “*” indicate compounds not confirmed based on a standard but having high confidence in its identity, while those ending in “**” indicate compounds for which a standard is not available, but for which there is reasonable confidence in its identity or the information provided.

In the inter-omic interaction analysis for metabolomics and clinical lab measures, those pairs significant in Arivale and measured in the TwinsUK were tested along with 965 additional pairs (5 chemistries by 193 metabolites) containing analyte groups frequently appearing in Arivale hits across experimental groups and related to bioenergetics and lipid metabolism (see Methods). Biologically young males showed the largest number of significant (pFDR < 0.1) hits, with 48 negatively modified associations mainly between phospholipids and LDL or total cholesterol and 3 positively modified associations, though low numbers of biologically younger males seemed to drive these associations. Biologically older males showed a negatively modified association between LDL and palmitoyl-linoleoyl-glycerol (16:0/18:2) [2]*. Biologically older females showed a negative modification on the association between glucose and isovalerylcarnitine (C5). APOE E2 positively modified the association between HDL and trimethylamine N-oxide but negatively modified the association between LDL and 1-palmitoyl-2-stearoyl-GPC (16:0/18:0) in females. The *APOE* ε2 allele, regardless of sex, significantly modified these same associations as well as negatively modified the associations between 1,2-dipalmitoyl-GPC (16:0/16:0) and both LDL and total cholesterol. Finally, in females APOE E4 positively modified the associations between isovalerylglycine and both total cholesterol and LDL; negatively modified the association between total cholesterol and sphingomyelin (d18:1/20:1, d18:2/20:0)*; and negatively modified the associations between blood glucose and both leucine and isoleucine. Representative modified associations are highlighted in Supplementary Figure 6 while full results are provided in Supplementary File 5.

With the differences in composition between the cohorts, no significant interactions in Arivale were able to be validated with FDR-significance. However there were a few interactions in TwinsUK having pre-adjusted *p* < 0.05 that corroborated with observed significant interactions in Arivale. The associations between glucose and choline, valine, aspartate, leucine, glutamate, and palmitoyl-linoleoyl-glycerol (16:0/18:2) [2]* were observed to be positively modified by biological oldness in males for both cohorts, all with pre-adjusted *p* < 0.02 (max pFDR of 0.37) in TwinsUK. In females, biological oldness positively modified the association between glucose and fructose with *p* = 0.015 (pFDR = 0.529) in TwinsUK. Increasing delta age in TwinsUK positively modified associations between triglycerides and both lactate (*p* = 3.00e-4, pFDR = 0.209) and glucose (*p* = 7.61e-3, pFDR = 0.474). Finally, APOE E2 positively modified the association between triglycerides and ribitol (*p* = 5.91e-3, pFDR = 0.349) in TwinsUK males.

## DISCUSSION

In this study, we leveraged two deeply phenotyped wellness cohorts of 2,229 (Arivale) and 1,696 (TwinsUK) individuals to analyze the systemic interplay between *APOE* genotype, delta age, sex, the blood metabolome, clinical chemistries, the proteome, and the microbiome. Our main findings include: (1) a resemblance between APOE E2 and E4-associated changes in blood metabolomics with increased DAG abundance, consistent with prior studies [23] and confirming APOE’s role in bioenergetics and potentially insulin resistance via altered lipid metabolism; (2) inter-omic associations in males and females are more similarly altered in a biologically older state than a biologically younger state, highlighting the importance of context-dependence; and (3) ‘omics associations between central bioenergetic analytes such as HbA1c, glucose, and glycolysis/TCA metabolites as well as lipids are similarly modified in APOE E2 and increased delta age, suggesting that *APOE* may systematically influence bioenergetic pathways, consistent with metabolic hypotheses of AD.

DAGs were among the most significant individual metabolites altered in APOE E2 and E4 in Arivale, and were positively associated in both groups as well as in the cohorts. DAGs have previously been shown to be increased in human plasma for ε2 carriers [23] and are here observed to be increased in ε4 carriers (pre-FDR-adjustment). In contrast, DAGs have also been previously shown to be decreased in ε4 carriers in the entorhinal cortex in mice models [22]. This discrepancy of APOE E4’s potential influence on DAG levels between the blood and brain may point to differences in DAG transport and sequestering as well as metabolism across APOE, or potentially highlight the limitations of mouse models to accurately reflect humans. More likely, differences in the brain from the periphery may be due to the fact that the brain only has a single particle system for re-distributing and off-loading cholesterol (APOE), while the periphery has a two-particle system: APOB for distributing cholesterol to cells, and APOA1, which helps transport excess cholesterol back to the liver [33, 34]. DAGs have also been observed to increase in both the plasma and the neocortex of AD patients, relative to controls [35]. DAGs are a major hallmark of overall lipid oxidation, indicative of lipase acting on triglycerides. DAGs act as secondary messengers to activate protein kinase C (PKC) and thus propel cascades producing reactive oxygen species (ROS) and inflammatory cytokines [36], are associated with insulin resistance [36–38], and have been suggested as biomarkers for sustained immune activation [35]. However, it is worth noting that different DAGs may have different effects based on the different isoforms and acyl groups present. A recent lipidomic study found overall plasma DAG levels to be positively correlated with higher steady-state plasma glucose levels, indicative of insulin resistance, yet also found DAGs to be negatively associated with age in participants with insulin resistance [39]. DAG oil has also been proposed as a therapeutic against metabolic syndrome and shown to lower serum insulin [40, 41]. High associations between plasma DAG levels and APOE isoforms suggests the lipid metabolism modulation by E2 (and potentially E4) increases plasma DAG accumulation, thereby potentially influencing insulin sensitivity and glucose uptake, even in a wellness state.

The similar pattern of elevated DAG species in both E2 and E4 is unexpected, given their typically opposing effects on aging in later decades of life. One reason may be that DAGs containing different acyl groups were variably associated with APOE E2 and E4. E2 was strongly associated with DAGs with palmitoyl and oleoyl residues. Palmitic and oleic acids are the most common saturated and monounsaturated fatty acids, respectively, and can both be synthesized *de novo* in humans or obtained via the diet, palmitic acid through meat and dairy or palm oils, and oleic acid largely from olive oil [42, 43]. Generally, increased palmitic acid is associated with poor health outcomes including inflammation, insulin resistance, and mitochondrial dysfunction [42], whereas oleic acid combats these effects and is associated with a healthier profile [43]. More broadly, increased levels of circulating fatty acids related to *de novo* lipogenesis are associated with increased T2D incidence [44]. E4s on the other hand tended to be more associated, albeit pre-FDR-adjustment, with those containing linoleoyl groups. Linoleic acid is the most commonly consumed polyunsaturated fatty acid, obtained exclusively in the diet, largely from vegetable oils [45]. Some overlapping DAG species were however associated with both E2 and E4. This similarity might be due to the APOE isoforms distinct transport mechanisms, with E4 preferentially binding larger fat particles such as very low-density lipoprotein (VLDL) and increased binding affinity to low density lipoprotein receptor, contrasting with E2 [46–48]. Both alleles might disrupt lipid transport or metabolism, leading to increased DAG as a shared feature of inefficiency. Additionally, while E2 is generally seen as beneficial and E4 as harmful, their effects are complex and not strictly opposite. For instance, E2 is linked to certain vascular and cervical disorders, while E4 offers some protection against diseases like type 2 diabetes (T2D) and obesity [49], and these roles vary by sex and ancestry. There may also be an age-dependent effect. In the CA-stratified metabolite abundance analysis in Arivale, three DAGs were significantly associated with APOE E2 in the oldest tertile yet two DAGs were significantly associated with APOE E4 in the youngest tertile, with no other DAGs having pFDR < 0.1 in any tertile. Further research is needed to understand these mechanisms.

Other age-dependent effects, including the variance of biological aging and delta age across lifespan, may have been difficult to detect in CA-stratified metabolomic abundance analyses due to reduced statistical power. Many of the metabolites associating generally with decelerated and accelerated biological aging regardless of CA in a non-stratified analysis lost significance when confining the sample size to a tertile CA range. There was a large degree of agreement in the associations for many metabolites across the CA tertiles, with no associations having pre-adjusted *p* < 0.05 in the overall analysis reversing sign within any CA tertile for the delta age status analysis, suggesting the possibility of signatures of accelerated or decelerated aging common across any CA. However, the presence of some metabolites significantly associated with the biologically old status within CA tertiles but not the non-stratified analysis, as well as the discordant significant associations of 1-linolenoyl-GPC (18:3)* and indoleacetate across CA tertiles suggest that elements of accelerated aging manifest differently across CA. Establishing the utility of the delta age metric may benefit from further study and characterization.

Our finding that 1-methylhistidine was significantly negatively associated with the biologically young but positively associated with the biologically old is interesting as a recent study identified the importance of histidine methylation in a subunit of mitochondrial complex I, NDUFB3, by METTL9 methyltransferase [50]. Mitochondrial complex I activity and production of ROS has been studied in the context of longevity and neurodegenerative disorders including Parkinson’s and Alzheimer’s [51–54]. Paradoxically, partial inhibition of mitochondrial complex I by the compound CP2 is beneficial for APP/PS1 mice that accumulate amyloid, restoring their cognitive function, as well as other markers of pathology, while treatment with CP2 in mice control animals shows no significant improvement [55]. This is consistent with the directionality of our observation of 1-methylhistidine being associated with increasing delta age, thus suggesting decreased METTL9 activity producing 1-methylhistidine and activating mitochondrial complex I is beneficial and associated with a lower delta age.

Plasmalogens, a subclass of glycerophospholipids found in high amounts in the brain, heart, and myelin, were enriched in positive associations with E2 and biologically older individuals as well as in negative associations for E4 and biologically younger individuals. This pattern with *APOE* is consistent with prior studies, the known plasmalogen level decrease in AD, and protective effect of plasmalogens against AD [56–58]. However, the associations with delta age seem contradictory to the known decrease with aging [57, 59]. This could be explained by the U-shaped pattern of plasmalogen abundance throughout aging, with plasmalogens increasing until age 30–40 to a plateau and then decreasing with age in the elderly after around age 70 [59]. With ~98% of the Arivale cohort being younger than 70, it is likely that plasmalogens would not yet show the decrease associated with the elderly, and higher levels would correspond to greater CA and BA. Similarly, the CA tertile-stratified individual metabolomic abundance analyses would likely not capture the effect due the lowest tertile (18–43 years) encompassing both the age range of expected increase and plateauing of plasmalogen levels and the greatest tertile (53–87 years) encompassing both the plateau and decrease, as well as the analysis having reduced statistical power.

Similarities in the constructed multi-omic atlases provide important insight as well. For example, the inter-omic association signatures of biologically older males and females are highly similar in contrast to the lack of similarity between biologically younger males and females. This implies that male and female ‘omics are more closely related in a state of perturbed health or accelerated aging than in a healthy state, which has been suggested previously [60]. One reason for this may be the impact of sex hormones, as sex-specific testosterone and estradiol decrease with age in males and females, respectively, while luteinizing hormone and follicle stimulating hormone increase with age in both sexes [61]. The specific inter-omic associations that were strengthened by biological oldness in both sexes seemed potentially indicative of metabolic imbalance such as insulin resistance or diabetes, which testosterone and estrogen protect against [62, 63]. For instance HbA1c was more strongly associated with central carbohydrates pyruvate and mannose. Increased glucose was more strongly associated with increased plasma (soluble) CD163, which is a marker of inflammation and associated with the development of T2D [64], as well as with increased plasma HGF, which is an inflammation regulator shown to be increased in chronic disease of several organs [65]. This trend is continued when analyzing delta age, seeing positively modified associations between glucose or HbA1c with several glycolysis and TCA metabolites including 1,5-AG, pyruvate, lactate, aconitate (cis or trans), and alpha-ketoglutarate. Of note, the interaction signatures of APOE E2 were similar to those of increased biological age. Four exactly overlapping associations were significant after multiple hypothesis correction in both male APOE E2s and biologically older males, including both HbA1c and glucose being more positively associated with phenol sulfate, a gut microbiome-produced uremic metabolite linked to albuminuria in diabetes and kidney disease [66, 67]. The other two positively modified associations were between hydroxyasparagine** and *Megasphaera*, and between FST and laureate (12:0), both also potentially highlighting an imbalance of bioenergetic pathways. Increased hydroxyasparagine abundance has been correlated to reduced kidney function [68], and though *Megasphaera* is among the butyrate-producing microbes generally contributing toward improved glucose homeostasis [69–71], *Megasphaera* abundance was found in one study to be increased in diabetics and associated with a higher fasting glucose [72], and in another to be increased in diabetic peripheral neuropathy and associated with higher a HOMA-IR [73]. Increased plasma FST is associated with increased T2D risk [74] and chronic kidney disease [75], though laureate (12:0) seems protective against insulin resistance, however [76]. Similar to the signature of biological oldness and increased delta age as well, *APOE* ε2 exhibited positively modified associations between HbA1c and TCA metabolites fumarate and maleate, as well as between glucose and TCA metabolite aconitate (cis or trans). Along this theme, some other significantly modified associations in APOE E2 males included the strengthened association between glucose and *Klebsiella*, a genus indicative of imbalance in the gut microbiome and known to modify the metabolome [77], and between triglycerides and ribitol, which disrupts central bioenergetic pathways via shifting the balance of metabolites participating in the TCA cycle, ultimately increasing glycolysis while decreasing oxidative phosphorylation [78].

These strengthened associations in APOE E2 suggest a rewiring of bioenergetic pathways reflective of accelerated aging, such as decreased sugar catabolism, potentially by shifting from glucose to fatty acid oxidation as a source of acetyl-CoA feeding into the citric acid cycle, or an increased conversion of sugars to HbA1c in the blood. This could signify that clinically well APOE E2 individuals exhibit a signature similar to insulin resistance as compared to E3, which may be supported by APOE E2’s association with increased DAGs, especially in older individuals, discussed earlier. This may be indicative of E2 showing decreased preference of glucose as an energy source as compared to E3, and thus have less insulin signaling in general. Because deregulated nutrient sensing is a hallmark of aging with insulin signaling decreasing in both physiological and accelerated aging [1], it is unsurprising that associations suggesting insulin resistance are found in biologically older individuals. As commented upon, this connection between APOE E2 and biological oldness seems contradictory, with APOE E2 generally predicting longevity and being protective against AD, whereas insulin resistance and diabetes as suggested here are risk factors for dementia and accelerated aging [79–81]. However, there may be an age-dependent effect of *APOE*, with APOE E2 imparting disadvantageous effects earlier in life while expanding longevity later. APOE E2 is associated with type III hyperlipoproteinemia [82, 83], has been linked to increased malaria infections and severity in early childhood [46], and has been observed to be associated with reduced reproductive efficiency [84]. Difficulties have been observed in breeding APOE E2 mice models [85], and female APOE E2 mice display an age-associated decreased insulin signaling in the hippocampus [86]. On the other hand, APOE E4 shows some advantages at earlier life stages in comparison to E3 such as improved neural and cognitive development in youth and decreased infant and perinatal mortality [46], and was recently found to have a protective effect against obesity and T2D [49]. This age-specific effect is an important consideration because cohort participants are relatively young and their health is representative of the US population. Therefore, E2 likely is not yet exhibiting its late-life advantages. Further, APOE E2’s potential association with insulin resistance from this analysis could suggest one of its mechanisms for supporting longevity, as a constitutive decrease in insulin signaling and insulin-like growth factor signaling would decrease the rate of cell growth and metabolism and thus reduce the rate of associated cellular damage seen in aging and AD [1].

TwinsUK was chosen as a validation cohort because of its similarities with Arivale in being composed of community dwelling individuals and the shared usage of the Metabolon platform, enabling more direct comparison of metabolomics data. Taken together, the two cohorts substantiated several plasma metabolite associations, including increased DAG in APOE E2 and E4; decreased cholesterol in E2; and decreased sphingomyelins in E2 (Table 3). The cholesterol finding is supported by the literature [58, 84] and aligns with increased cholesterol being a risk factor for AD [87], though plasma cholesterol was observed to be increased and associated with age in mouse models of APOE E2 [88]. The lower levels of plasma sphingomyelins observed in APOE E2 in this study may be indicative of a protective effect of E2, as increased serum sphingomyelin species are associated with worse biomarkers and clinical measures of AD [89]. Though lower levels of sphingomyelins in the blood are beneficial, reduced sphingomyelin levels in the brain are detrimental, with AD brains exhibiting reduced sphingomyelin levels [90, 91] with reductions more pronounced in APOE E4 as compared to other isoforms [92]. Further, APOE E4 mouse model brains displayed reduced sphingomyelin levels in both the entorhinal cortex and primary visual cortex, regions vulnerable and resistant to AD, respectively [22].

However, we were unable to reproduce some of our other Arivale findings in TwinsUK, including the similarities between APOE E2 and biological oldness in males, due to data limitations such as a small male sample size (maximum *n* = 55 for the tests of male APOE E2 and biological old males), giving low statistical power to some models and inflating the number of significant hits in biologically younger males. Some results observed in Arivale, such as biological oldness positively modifying associations between central bioenergetic metabolites and APOE E2 positively modifying the association between triglycerides and ribitol in males, trended in the same direction, however significance was lost after FDR correction. Validation results for the interaction analysis are thus uncertain. Even so, other FDR-significant interactions in TwinsUK were identified, including altered lipidomic associations in APOE E2 and in APOE E4 in females, confirming *APOE* exerts pressure on metabolic pathways and associations with the potential of ‘rewiring’ them to influence health status overall.

Limitations of this study include the use of cross-sectional data with no available disease or longevity outcomes to analyze. Metabolomics data was also limited to the plasma, not allowing further study on transport and localized measures such as brain metabolomics. Lifestyle factors such as diet, exercise, and medication use other than cholesterol reducing drugs were not analyzed. The available cohorts were also predominantly composed of non-Hispanic Whites (71% in Arivale, >99% in TwinsUK), which limits the generalizability of the aforementioned significant differences in *APOE*’s manifestation across ethnicities. Survivorship bias is another potential limitation to results for APOE E4 associations, as it is well documented that older ε4 carriers represent a cognitively resilient population because many ε4 carriers die prematurely relative to ε3/ε3 individuals [93–96]. Validation was limited by data differences between the Arivale and TwinsUK cohort, including a lower percentage and sample size of males in TwinsUK (3.6%, *n* = 61 unique individuals); lack of significant DAG species in the validation set; and lack of HbA1c measures in the validation set, having only 33 total measurements, all in females and not enough to allow all model covariates to be represented. While this study provides promising preliminary findings, future studies with greater statistical power, more diverse participants, and longitudinal data are needed to understand the universality of these results, further examine age-dependent effects of *APOE* and biological age, and assess the effectiveness of related interventions. In addition to validations in other populations, further studies validating *APOE*’s influence on specific metabolites and pathways *in vitro* or in mice or other animal models would be valuable.

These findings substantiate *APOE*’s influence on bioenergetic metabolism, show agreement with current understanding and hypotheses of *APOE* including context dependencies such as sex differences, and suggest a mechanism for *APOE*-associated longevity and potentially AD pathology. Further, the results provide a preliminary atlas of inter-omic associations useful for possible interventions to offset *APOE*-associated risk in the prodromal stages of AD and cardiovascular disease and to extend healthspan.

## METHODS

### Arivale wellness cohort and data collection

Research subjects in this study were voluntary, anonymous participants of the Arivale Scientific Wellness program described by Zubair et al. [26]. The program aimed to leverage the collection of dense health data from subscribers to offer personalized wellness coaching from a systems biology perspective. The collection of plasma metabolomics (Metabolon platform), plasma proteomics (Olink platform), microbiomics (16S V4 amplicon sequencing data from stool samples), and clinical chemistries data has been described thoroughly in Wilmanski et al. [97]. In this study, only individuals with whole genome sequencing were included. APOE status was determined from single nucleotide polymorphisms (SNPs) from this data, with both homozygotes for (ε2/ε2) and carriers of (ε2/ε3) the ε2 allele being defined as APOE E2; ε3/ε3 being defined as APOE E3; and both ε3/ε4 and ε4/ε4 being defined as APOE E4. ε2/ε4 individuals were excluded from analysis. The dataset contained no ε1 alleles.

### TwinsUK cohort and data collection

The TwinsUK cohort was originally intended to investigate rheumatologic diseases in identical twins in the United Kingdom, and has since expanded to encompass over 15,000 volunteer identical and non-identical twins [27]. Similar to the Arivale cohort, the voluntary participants are community dwelling, representative of the health of the population, and deeply phenotyped. Unlike in Arivale, no coaching or intervention is performed. For this study, only the 1696 individuals with metabolomics and genotyping data were included. APOE status was determined from SNPs from Illumina assays. As in the Arivale cohort, both homozygotes for (ε2/ε2) and carriers of (ε2/ε3) the ε2 allele were defined as APOE E2; ε3/ε3 were defined as APOE E3; and both ε3/ε4 and ε4/ε4 were defined as APOE E4. ε2/ε4 individuals were recorded but excluded from analysis, and the dataset contained no ε1 alleles. Metabolomics data was obtained using the Metabolon platform, the same platform as Arivale, and has been described previously in Long et al. [28]. Demographic differences for the Arivale and TwinsUK individuals at baseline are summarized in Table 1.

### Biological age and delta age

Biological age (BA) values for the Arivale dataset were previously calculated by Earls et al. [3]. Briefly, four baseline biological age measures were computed: one from clinical labs, another from proteomics, one from metabolomics, and one combining the first three sources. Each of the models were obtained utilizing the Klemera-Doubal method, and were constructed separately for males and females. In this study, the average of the clinical lab and proteomic BA was used for metabolomic analyses, and the combined measure of BA was used for multi-omic analyses. Chronological age (CA) at baseline was subtracted from BA to yield ‘delta age’. A delta age of over 7.5 years (about one standard deviation for both male and female, 7.8 years for female, 8.5 years for male) was treated as ‘biologically older’, and a delta age of less than −7.5 years was taken to be ‘biologically younger’. Delta age was not significantly different across APOE status (Supplementary Figure 2A), and sorting of APOE and delta age groups was not interdependent based on Chi2 testing (Supplementary Figure 2B).

BA and delta age values for the TwinsUK cohort were calculated using the same modeling method used by Earls et al. [3] to calculate a metabolomics-based BA in the Arivale cohort. The 494 metabolites overlapping out of the 740 appearing in the original model were used following the same Klemera-Doubal (KD) method implemented by Earls to train another model with the TwinsUK data: BA was predicted for each sample by taking the average of ten iterations of ten-fold cross-validation, training the model separately for males and females. CA was then subtracted from BA to yield delta age. Retraining a new model independently for TwinsUK avoids errors due to batch effects. Similar to Arivale, a delta age of over 7.5 years was treated as ‘biologically older’, and a delta age of less than −7.5 years was taken to be ‘biologically younger’ for females (delta age standard deviation for females 7.8 years), however the delta age cutoff for males was set to +/− 5.0 years to better reflect the standard deviation for males (5.1 years) and smaller sample size (Supplementary Figure 3). For TwinsUK, delta age was significantly different across APOE status (Supplementary Figure 4).

### Differential metabolite abundance analysis

For individual baseline metabolite level comparisons in Arivale, 896 winsorized metabolites from the Metabolon platform were analyzed after excluding those with more than 20% missingness. The presence of asterisks “*” in metabolite names given by the Metabolon platform indicates the metabolites status corresponding to Metabolomics Standards Initiative Tier 1 identification [98]. A name with no asterisk represents Tier 1 identification, while one asterisk indicates a compound that has not been confirmed based on a standard but for which there is high confidence in its identity (not Tier 1), and two asterisks indicate a compound for which a standard is not available, but for which there is reasonable confidence in its identity or the information provided (not Tier 1). Note that isomer information for lipids such as DAGs in the Metabolon platform are not known, for example the metabolite reported as linoleoyl-arachidonoyl-glycerol (18:2/20:4) [1]* is a DAG having linoleic and arachidonic acid residues, however their position relative to the glycerol is ambiguous. Triacylglyceride species were also not available for analysis in the Metabolon platform. Missing data was replaced via random forest imputation, which has shown to be effective for liquid chromatography-mass spectrometry derived metabolomics data [99]. Metabolomics data was then log2 transformed, and then z-scored. For GLMs analyzing differential metabolite abundance as reported in Figure 2, the model, log2(z-scored metabolite) = intercept + **APOE E2 + APOE E4** + age + sex(Male) + body mass index (BMI) + cholesterol meds(self-reported) + (genetics) Principal Component (PC)1 + PC2 + e, was used for analyzing APOE, and the model, log2(z-scored metabolite) = intercept + **Biologically Young + >Biologically Old** + age + sex(Male) + BMI + cholesterol meds(self-reported) + PC1 + PC2 + e, was used for analyzing delta age status (analyzed *β*-coefficients bolded). The analysis was then repeated within CA tertiles. The first two PCs used in the model were previously calculated [97]. The PCs display a non-significant difference across APOE E2, E3, and E4 statuses in the subset of Arivale used in the differential metabolite abundance analyses (Kruskal-Wallis test, *p* = 0.168 and *p* = 0.248 for PC1 and PC2, respectively).

For TwinsUK, 752 metabolites also from the Metabolon platform remained after excluding those with more than 20% missingness. After random forest imputation, the earliest visit measurement for each individual was used after removing samples recorded as non-fasting. The following GLM model was used for analyzing APOE: log2(z-scored metabolite) = intercept + **APOE E2 + APOE E4** + age + sex (Male) + BMI + cholesterol-reducing medications(self-reported) + batch (2–5 compared to 1) + e (analyzed *β*-coefficients bolded). The analysis was then repeated within CA tertiles. Delta age was not analyzed because BA models in TwinsUK were solely derived from metabolomics.

Following GLMs, an enrichment analysis of the sub-pathways annotated in the Metabolon platform was performed both on the sets of metabolites with significant (pFDR < 0.1) and pre-adjusted (*p* < 0.05) positive and negative associations for each experimental group. A standard overrepresentation test was performed for the enrichment analysis, using a hypergeometric distribution model with a survival function to calculate *p*-values for each sub-pathway for each set of associations.

### Inter-omic interaction analysis

For the analysis of inter-omic interactions with APOE and health, individuals were stratified by sex and then again by either APOE status or delta age status to offer direct comparisons, creating eight subsets.

For Arivale, baseline metabolomic, proteomic, and clinical chemistries data were winsorized for use in the analysis via iteratively shrinking outliers to within five standard deviations of the median. Proteomic data was from the Olink platform. Clinical chemistries were limited to only those from the Laboratory of Cell Analysis, and individuals using a different platform were dropped. Microbiome data was from DNA Genotek OMNIgene GUT collection kits sequenced by Second Genome and DNA Genotek. Baseline microbiome data was centered log-ratio transformed and filtered for rare taxa using mean and prevalence thresholds of 10 and 0.1, respectively. A total of 509,360 inter-omic combinations of analytes were tested from 876 metabolites, 274 proteins, 67 clinical draws, and 158 microbiome genera having less than 20% missing values. Those analyte pairs with significant (pFDR < 0.1) interaction results in Arivale were tested for validation in the TwinsUK cohort, given data availability. The inter-omic interactions between Glucose, LDL, HDL, Triglycerides, and Total Cholesterol from the clinical chemistries and metabolites in the ‘TCA Cycle’, ‘Glycolysis, Gluconeogenesis, and Pyruvate Metabolism’, ‘Fructose, Mannose and Galactose Metabolism’, ‘Pentose Metabolism’, ‘Oxidative Phosphorylation’, ‘Phospholipid Metabolism’, ‘Sphingolipid Metabolism’, ‘Leucine, Isoleucine and Valine Metabolism’, or ‘Diacylglycerol’ subpathways (Metabolon labeling) were additionally analyzed in the validation cohort. TwinsUK data preprocessing followed the same method as in Arivale, with a 20% missingness threshold and iterative winsorization of outliers to within 5 standard deviations of the median. All samples indicating non-fasting were dropped, and the TwinsUK data from the earliest visit containing values for both clinical test and metabolite for each individual were used in the analysis. Intra-omic combinations were not tested in either cohort.

GLMs were performed for each subset with the following model for Arivale: analyte1 = intercept + analyte2 + X + analyte2*X + age + season(reference = Fall) + BMI + cholesterol meds(self-reported) + (genetics) Principal Component (PC)1 + PC2 + e, where X is the experimental group analyzed (ie: APOE E2 or E4, or Biologically Young or Old statuses) and ‘analyte2*X’ represents the interaction term between the second analyte and the experimental group, bolded here to indicate it is the *β*-coefficient analyzed. The first two PCs used in the model were previously calculated [97]. For TwinsUK, the model was: clinical test = intercept + metabolite + X + metabolite*X + age + BMI + cholesterol meds(user) + e. Each experimental group was analyzed separately and stratified by sex to isolate and narrow focus on the variable of interest. An additional set of models was tested in each cohort as well, with sex as a covariate instead of a stratified variable, and the ‘experimental groups’ being ε2 or ε4 allele dosage (with possible values being 0, 1, or 2) or delta age value in days. The relatively small number of analyte pairs in the GLMs failing with a ‘NaN, inf or invalid value detected in weights, estimation infeasible’ error, were noted but ignored.

All GLM models in statistical analysis assumed a gaussian distribution with an identity link and were set at 2000 maximum iterations. For the inter-omic interaction analysis, if the first analyte exhibited a skew of greater magnitude than 1.5, a gamma distribution was used with a log link instead, with values of zero being replaced with half the minimum non-zero value. FDR significance was determined by adjusting *p*-values corresponding to APOE and health statuses by the Benjamini-Hochberg method with the FDR set to 5% [100]. GLMs in the metabolite abundance and inter-omic interaction analysis were performed using the glm function from the statsmodels package version 0.13.0 in Python version 3.9.7.

### Data and code availability

The Arivale datasets used in this study are not publicly available owing to both ethical and legal reasons, but qualified researchers can request access to the de-identified datasets for research purposes through a Data Use Agreement. Inquiries about data access should be sent to data-access@isbscience.org and will be responded to within seven business days. The TwinsUK datasets used in this study were provided by the Department of Twin Research and Genetic Epidemiology (King’s College London) after the approval of our Data Access Application (project number E1199). Requests should be referred to their website (http://twinsuk.ac.uk/resources-for-researchers/access-our-data/). Code used in this study is freely available on GitHub (https://github.com/PriceLab/APOE-Multiomics).

## Supplementary Materials

Supplementary Figures

Supplementary Tables

Supplementary File 1

Supplementary File 2

Supplementary File 3

Supplementary File 4

Supplementary File 5

## References

[1] López-Otín C, Blasco MA, Partridge L, Serrano M, Kroemer G. Hallmarks of aging: An expanding universe. Cell. 2023; 186:243–78. 10.1016/j.cell.2022.11.001 36599349

[2] DeTure MA, Dickson DW. The neuropathological diagnosis of Alzheimer's disease. Mol Neurodegener. 2019; 14:32. 10.1186/s13024-019-0333-5 31375134 PMC6679484

[3] Earls JC, Rappaport N, Heath L, Wilmanski T, Magis AT, Schork NJ, Omenn GS, Lovejoy J, Hood L, Price ND. Multi-Omic Biological Age Estimation and Its Correlation With Wellness and Disease Phenotypes: A Longitudinal Study of 3,558 Individuals. J Gerontol A Biol Sci Med Sci. 2019; 74:S52–60. 10.1093/gerona/glz220 31724055 PMC6853785

[4] Levine ME. Modeling the rate of senescence: can estimated biological age predict mortality more accurately than chronological age? J Gerontol A Biol Sci Med Sci. 2013; 68:667–74. 10.1093/gerona/gls233 23213031 PMC3660119

[5] Klemera P, Doubal S. A new approach to the concept and computation of biological age. Mech Ageing Dev. 2006; 127:240–8. 10.1016/j.mad.2005.10.004 16318865

[6] Strittmatter WJ, Saunders AM, Schmechel D, Pericak-Vance M, Enghild J, Salvesen GS, Roses AD. Apolipoprotein E: high-avidity binding to beta-amyloid and increased frequency of type 4 allele in late-onset familial Alzheimer disease. Proc Natl Acad Sci U S A. 1993; 90:1977–81. 10.1073/pnas.90.5.1977 8446617 PMC46003

[7] Mahley RW, Weisgraber KH, Huang Y. Apolipoprotein E4: a causative factor and therapeutic target in neuropathology, including Alzheimer's disease. Proc Natl Acad Sci U S A. 2006; 103:5644–51. 10.1073/pnas.0600549103 16567625 PMC1414631

[8] Safieh M, Korczyn AD, Michaelson DM. ApoE4: an emerging therapeutic target for Alzheimer's disease. BMC Med. 2019; 17:64. 10.1186/s12916-019-1299-4 30890171 PMC6425600

[9] Li Z, Shue F, Zhao N, Shinohara M, Bu G. APOE2: protective mechanism and therapeutic implications for Alzheimer's disease. Mol Neurodegener. 2020; 15:63. 10.1186/s13024-020-00413-4 33148290 PMC7640652

[10] Reiman EM, Arboleda-Velasquez JF, Quiroz YT, Huentelman MJ, Beach TG, Caselli RJ, Chen Y, Su Y, Myers AJ, Hardy J, Paul Vonsattel J, Younkin SG, Bennett DA, et al, and Alzheimer’s Disease Genetics Consortium. Exceptionally low likelihood of Alzheimer's dementia in APOE2 homozygotes from a 5,000-person neuropathological study. Nat Commun. 2020; 11:667. 10.1038/s41467-019-14279-8 32015339 PMC6997393

[11] Shinohara M, Kanekiyo T, Tachibana M, Kurti A, Shinohara M, Fu Y, Zhao J, Han X, Sullivan PM, Rebeck GW, Fryer JD, Heckman MG, Bu G. *APOE2* is associated with longevity independent of Alzheimer's disease. Elife. 2020; 9:e62199. 10.7554/eLife.62199 33074098 PMC7588231

[12] Sebastiani P, Gurinovich A, Nygaard M, Sasaki T, Sweigart B, Bae H, Andersen SL, Villa F, Atzmon G, Christensen K, Arai Y, Barzilai N, Puca A, et al. APOE Alleles and Extreme Human Longevity. J Gerontol A Biol Sci Med Sci. 2019; 74:44–51. 10.1093/gerona/gly174 30060062 PMC6298189

[13] Demetrius LA, Driver J. Alzheimer's as a metabolic disease. Biogerontology. 2013; 14:641–9. 10.1007/s10522-013-9479-7 24249045

[14] Caldwell CC, Yao J, Brinton RD. Targeting the prodromal stage of Alzheimer's disease: bioenergetic and mitochondrial opportunities. Neurotherapeutics. 2015; 12:66–80. 10.1007/s13311-014-0324-8 25534394 PMC4322082

[15] Liu PP, Xie Y, Meng XY, Kang JS. History and progress of hypotheses and clinical trials for Alzheimer's disease. Signal Transduct Target Ther. 2019; 4:29. 10.1038/s41392-019-0063-8 31637009 PMC6799833

[16] Huo Z, Yu L, Yang J, Zhu Y, Bennett DA, Zhao J. Brain and blood metabolome for Alzheimer's dementia: findings from a targeted metabolomics analysis. Neurobiol Aging. 2020; 86:123–33. 10.1016/j.neurobiolaging.2019.10.014 31785839 PMC6995427

[17] Wolf AB, Caselli RJ, Reiman EM, Valla J. APOE and neuroenergetics: an emerging paradigm in Alzheimer's disease. Neurobiol Aging. 2013; 34:1007–17. 10.1016/j.neurobiolaging.2012.10.011 23159550 PMC3545040

[18] Johnson LA. APOE and metabolic dysfunction in Alzheimer's disease. Int Rev Neurobiol. 2020; 154:131–51. 10.1016/bs.irn.2020.02.002 32739002

[19] Roy ER, Chiu G, Li S, Propson NE, Kanchi R, Wang B, Coarfa C, Zheng H, Cao W. Concerted type I interferon signaling in microglia and neural cells promotes memory impairment associated with amyloid β plaques. Immunity. 2022; 55:879–94.e6. 10.1016/j.immuni.2022.03.018 35443157 PMC9109419

[20] Roy ER, Wang B, Wan YW, Chiu G, Cole A, Yin Z, Propson NE, Xu Y, Jankowsky JL, Liu Z, Lee VM, Trojanowski JQ, Ginsberg SD, et al. Type I interferon response drives neuroinflammation and synapse loss in Alzheimer disease. J Clin Invest. 2020; 130:1912–30. 10.1172/JCI133737 31917687 PMC7108898

[21] Heath L, Earls JC, Magis AT, Kornilov SA, Lovejoy JC, Funk CC, Rappaport N, Logsdon BA, Mangravite LM, Kunkle BW, Martin ER, Naj AC, Ertekin-Taner N, et al, and Alzheimer’s Disease Genetics Consortium. Manifestations of Alzheimer's disease genetic risk in the blood are evident in a multiomic analysis in healthy adults aged 18 to 90. Sci Rep. 2022; 12:6117. 10.1038/s41598-022-09825-2 35413975 PMC9005657

[22] Miranda AM, Ashok A, Chan RB, Zhou B, Xu Y, McIntire LB, Area-Gomez E, Di Paolo G, Duff KE, Oliveira TG, Nuriel T. Effects of APOE4 allelic dosage on lipidomic signatures in the entorhinal cortex of aged mice. Transl Psychiatry. 2022; 12:129. 10.1038/s41398-022-01881-6 35351864 PMC8964762

[23] Sebastiani P, Song Z, Ellis D, Tian Q, Schwaiger-Haber M, Stancliffe E, Lustgarten MS, Funk CC, Baloni P, Yao CH, Joshi S, Marron MM, Gurinovich A, et al. A metabolomic signature of the APOE2 allele. Geroscience. 2023; 45:415–26. 10.1007/s11357-022-00646-9 35997888 PMC9886693

[24] Arboleda-Velasquez JF, Lopera F, O'Hare M, Delgado-Tirado S, Marino C, Chmielewska N, Saez-Torres KL, Amarnani D, Schultz AP, Sperling RA, Leyton-Cifuentes D, Chen K, Baena A, et al. Resistance to autosomal dominant Alzheimer's disease in an APOE3 Christchurch homozygote: a case report. Nat Med. 2019; 25:1680–3. 10.1038/s41591-019-0611-3 31686034 PMC6898984

[25] Paterson T, Rohrs J, Hohman TJ, Mapstone M, Levey AI, Hood L, Funk CC, and Alzheimer’s Disease Neuroimaging Initiative. Multi-omic ADNI CSF and plasma data integration identifies distinct metabolic transitions in disease progression in Alzheimer’s Disease. bioRxiv. 2024. 10.1101/2024.07.23.604835

[26] Zubair N, Conomos MP, Hood L, Omenn GS, Price ND, Spring BJ, Magis AT, Lovejoy JC. Genetic Predisposition Impacts Clinical Changes in a Lifestyle Coaching Program. Sci Rep. 2019; 9:6805. 10.1038/s41598-019-43058-0 31048771 PMC6497671

[27] Moayyeri A, Hammond CJ, Valdes AM, Spector TD. Cohort Profile: TwinsUK and healthy ageing twin study. Int J Epidemiol. 2013; 42:76–85. 10.1093/ije/dyr207 22253318 PMC3600616

[28] Long T, Hicks M, Yu HC, Biggs WH, Kirkness EF, Menni C, Zierer J, Small KS, Mangino M, Messier H, Brewerton S, Turpaz Y, Perkins BA, et al. Whole-genome sequencing identifies common-to-rare variants associated with human blood metabolites. Nat Genet. 2017; 49:568–78. 10.1038/ng.3809 28263315

[29] Juraschek SP, Steffes MW, Miller ER 3rd, Selvin E. Alternative markers of hyperglycemia and risk of diabetes. Diabetes Care. 2012; 35:2265–70. 10.2337/dc12-0787 22875225 PMC3476908

[30] Pramodkumar TA, Jayashri R, Gokulakrishnan K, Velmurugan K, Pradeepa R, Venkatesan U, Saravanan P, Uma R, Anjana RM, Mohan V. 1,5 Anhydroglucitol in gestational diabetes mellitus. J Diabetes Complications. 2019; 33:231–5. 10.1016/j.jdiacomp.2018.11.010 30594413

[31] Su Y, Chen D, Yuan D, Lausted C, Choi J, Dai CL, Voillet V, Duvvuri VR, Scherler K, Troisch P, Baloni P, Qin G, Smith B, et al, and ISB-Swedish COVID19 Biobanking Unit. Multi-Omics Resolves a Sharp Disease-State Shift between Mild and Moderate COVID-19. Cell. 2020; 183:1479–95.e20. 10.1016/j.cell.2020.10.037 33171100 PMC7598382

[32] Frick EA, Emilsson V, Jonmundsson T, Steindorsdottir AE, Johnson ECB, Puerta R, Dammer EB, Shantaraman A, Cano A, Boada M, Valero S, García-González P, Gudmundsson EF, et al. Serum proteomics reveal *APOE-ε4*-dependent and *APOE-ε4*-independent protein signatures in Alzheimer’s disease. Nat Aging. 2024; 4:1446–64. 10.1038/s43587-024-00693-1 39169269 PMC11485263

[33] Mehta A, Shapiro MD. Apolipoproteins in vascular biology and atherosclerotic disease. Nat Rev Cardiol. 2022; 19:168–79. 10.1038/s41569-021-00613-5 34625741

[34] Vance JE, Hayashi H. Formation and function of apolipoprotein E-containing lipoproteins in the nervous system. Biochim Biophys Acta. 2010; 1801:806–18. 10.1016/j.bbalip.2010.02.007 20170744

[35] Wood PL, Cebak JE, Woltjer RL. Diacylglycerols as biomarkers of sustained immune activation in Proteinopathies associated with dementia. Clin Chim Acta. 2018; 476:107–10. 10.1016/j.cca.2017.11.009 29146478

[36] Volpe CMO, Villar-Delfino PH, Dos Anjos PMF, Nogueira-Machado JA. Cellular death, reactive oxygen species (ROS) and diabetic complications. Cell Death Dis. 2018; 9:119. 10.1038/s41419-017-0135-z 29371661 PMC5833737

[37] Amati F. Revisiting the diacylglycerol-induced insulin resistance hypothesis. Obes Rev. 2012 (Suppl 2); 13:40–50. 10.1111/j.1467-789X.2012.01036.x 23107258

[38] Kolczynska K, Loza-Valdes A, Hawro I, Sumara G. Diacylglycerol-evoked activation of PKC and PKD isoforms in regulation of glucose and lipid metabolism: a review. Lipids Health Dis. 2020; 19:113. 10.1186/s12944-020-01286-8 32466765 PMC7257441

[39] Hornburg D, Wu S, Moqri M, Zhou X, Contrepois K, Bararpour N, Traber GM, Su B, Metwally AA, Avina M, Zhou W, Ubellacker JM, Mishra T, et al. Dynamic lipidome alterations associated with human health, disease and ageing. Nat Metab. 2023; 5:1578–94. 10.1038/s42255-023-00880-1 37697054 PMC10513930

[40] Yanai H, Tomono Y, Ito K, Furutani N, Yoshida H, Tada N. Diacylglycerol oil for the metabolic syndrome. Nutr J. 2007; 6:43. 10.1186/1475-2891-6-43 18072966 PMC2235882

[41] Yanai H, Yoshida H, Tomono Y, Hirowatari Y, Kurosawa H, Matsumoto A, Tada N. Effects of diacylglycerol on glucose, lipid metabolism, and plasma serotonin levels in lean Japanese. Obesity (Silver Spring). 2008; 16:47–51. 10.1038/oby.2007.46 18223611

[42] Carta G, Murru E, Banni S, Manca C. Palmitic Acid: Physiological Role, Metabolism and Nutritional Implications. Front Physiol. 2017; 8:902. 10.3389/fphys.2017.00902 29167646 PMC5682332

[43] Piccinin E, Cariello M, De Santis S, Ducheix S, Sabbà C, Ntambi JM, Moschetta A. Role of Oleic Acid in the Gut-Liver Axis: From Diet to the Regulation of Its Synthesis via Stearoyl-CoA Desaturase 1 (SCD1). Nutrients. 2019; 11:2283. 10.3390/nu11102283 31554181 PMC6835877

[44] Imamura F, Fretts AM, Marklund M, Ardisson Korat AV, Yang WS, Lankinen M, Qureshi W, Helmer C, Chen TA, Virtanen JK, Wong K, Bassett JK, Murphy R, et al, and InterAct Consortium. Fatty acids in the de novo lipogenesis pathway and incidence of type 2 diabetes: A pooled analysis of prospective cohort studies. PLoS Med. 2020; 17:e1003102. 10.1371/journal.pmed.1003102 32530938 PMC7292352

[45] Whelan J, Fritsche K. Linoleic acid. Adv Nutr. 2013; 4:311–2. 10.3945/an.113.003772 23674797 PMC3650500

[46] Huebbe P, Rimbach G. Evolution of human apolipoprotein E (APOE) isoforms: Gene structure, protein function and interaction with dietary factors. Ageing Res Rev. 2017; 37:146–61. 10.1016/j.arr.2017.06.002 28647612

[47] Tomaszewski N, He X, Solomon V, Lee M, Mack WJ, Quinn JF, Braskie MN, Yassine HN. Effect of APOE Genotype on Plasma Docosahexaenoic Acid (DHA), Eicosapentaenoic Acid, Arachidonic Acid, and Hippocampal Volume in the Alzheimer's Disease Cooperative Study-Sponsored DHA Clinical Trial. J Alzheimers Dis. 2020; 74:975–90. 10.3233/JAD-191017 32116250 PMC7156328

[48] Johnson LA, Olsen RH, Merkens LS, DeBarber A, Steiner RD, Sullivan PM, Maeda N, Raber J. Apolipoprotein E-low density lipoprotein receptor interaction affects spatial memory retention and brain ApoE levels in an isoform-dependent manner. Neurobiol Dis. 2014; 64:150–62. 10.1016/j.nbd.2013.12.016 24412220 PMC3936477

[49] Lumsden AL, Mulugeta A, Zhou A, Hyppönen E. Apolipoprotein E (APOE) genotype-associated disease risks: a phenome-wide, registry-based, case-control study utilising the UK Biobank. EBioMedicine. 2020; 59:102954. 10.1016/j.ebiom.2020.102954 32818802 PMC7452404

[50] Davydova E, Shimazu T, Schuhmacher MK, Jakobsson ME, Willemen HLD, Liu T, Moen A, Ho AYY, Małecki J, Schroer L, Pinto R, Suzuki T, Grønsberg IA, et al. The methyltransferase METTL9 mediates pervasive 1-methylhistidine modification in mammalian proteomes. Nat Commun. 2021; 12:891. 10.1038/s41467-020-20670-7 33563959 PMC7873184

[51] Flannery PJ, Trushina E. Mitochondrial dynamics and transport in Alzheimer's disease. Mol Cell Neurosci. 2019; 98:109–20. 10.1016/j.mcn.2019.06.009 31216425 PMC6614006

[52] Xiong N, Long X, Xiong J, Jia M, Chen C, Huang J, Ghoorah D, Kong X, Lin Z, Wang T. Mitochondrial complex I inhibitor rotenone-induced toxicity and its potential mechanisms in Parkinson's disease models. Crit Rev Toxicol. 2012; 42:613–32. 10.3109/10408444.2012.680431 22574684

[53] Sharma A, Smith HJ, Yao P, Mair WB. Causal roles of mitochondrial dynamics in longevity and healthy aging. EMBO Rep. 2019; 20:e48395. 10.15252/embr.201948395 31667999 PMC6893295

[54] Miwa S, Jow H, Baty K, Johnson A, Czapiewski R, Saretzki G, Treumann A, von Zglinicki T. Low abundance of the matrix arm of complex I in mitochondria predicts longevity in mice. Nat Commun. 2014; 5:3837. 10.1038/ncomms4837 24815183 PMC4024759

[55] Stojakovic A, Trushin S, Sheu A, Khalili L, Chang SY, Li X, Christensen T, Salisbury JL, Geroux RE, Gateno B, Flannery PJ, Dehankar M, Funk CC, et al. Partial inhibition of mitochondrial complex I ameliorates Alzheimer's disease pathology and cognition in APP/PS1 female mice. Commun Biol. 2021; 4:61. 10.1038/s42003-020-01584-y 33420340 PMC7794523

[56] Goodenowe DB, Senanayake V. Relation of Serum Plasmalogens and *APOE* Genotype to Cognition and Dementia in Older Persons in a Cross-Sectional Study. Brain Sci. 2019; 9:92. 10.3390/brainsci9040092 31022959 PMC6523320

[57] Senanayake V, Goodenowe DB. Plasmalogen deficiency and neuropathology in Alzheimer's disease: Causation or coincidence? Alzheimers Dement (N Y). 2019; 5:524–32. 10.1016/j.trci.2019.08.003 31650009 PMC6804645

[58] Wang T, Huynh K, Giles C, Mellett NA, Duong T, Nguyen A, Lim WLF, Smith AA, Olshansky G, Cadby G, Hung J, Hui J, Beilby J, et al. APOE ε2 resilience for Alzheimer's disease is mediated by plasma lipid species: Analysis of three independent cohort studies. Alzheimers Dement. 2022; 18:2151–66. 10.1002/alz.12538 35077012 PMC9787288

[59] Bozelli JC Jr, Azher S, Epand RM. Plasmalogens and Chronic Inflammatory Diseases. Front Physiol. 2021; 12:730829. 10.3389/fphys.2021.730829 34744771 PMC8566352

[60] Zimmer A, Korem Y, Rappaport N, Wilmanski T, Baloni P, Jade K, Robinson M, Magis AT, Lovejoy J, Gibbons SM, Hood L, Price ND. The geometry of clinical labs and wellness states from deeply phenotyped humans. Nat Commun. 2021; 12:3578. 10.1038/s41467-021-23849-8 34117230 PMC8196202

[61] Chahal HS, Drake WM. The endocrine system and ageing. J Pathol. 2007; 211:173–80. 10.1002/path.2110 17200939

[62] Yao QM, Wang B, An XF, Zhang JA, Ding L. Testosterone level and risk of type 2 diabetes in men: a systematic review and meta-analysis. Endocr Connect. 2018; 7:220–31. 10.1530/EC-17-0253 29233816 PMC5793809

[63] De Paoli M, Zakharia A, Werstuck GH. The Role of Estrogen in Insulin Resistance: A Review of Clinical and Preclinical Data. Am J Pathol. 2021; 191:1490–8. 10.1016/j.ajpath.2021.05.011 34102108

[64] Parkner T, Sørensen LP, Nielsen AR, Fischer CP, Bibby BM, Nielsen S, Pedersen BK, Møller HJ. Soluble CD163: a biomarker linking macrophages and insulin resistance. Diabetologia. 2012; 55:1856–62. 10.1007/s00125-012-2533-1 22450890

[65] Nakamura T, Mizuno S. The discovery of hepatocyte growth factor (HGF) and its significance for cell biology, life sciences and clinical medicine. Proc Jpn Acad Ser B Phys Biol Sci. 2010; 86:588–610. 10.2183/pjab.86.588 20551596 PMC3081175

[66] Kikuchi K, Saigusa D, Kanemitsu Y, Matsumoto Y, Thanai P, Suzuki N, Mise K, Yamaguchi H, Nakamura T, Asaji K, Mukawa C, Tsukamoto H, Sato T, et al. Gut microbiome-derived phenyl sulfate contributes to albuminuria in diabetic kidney disease. Nat Commun. 2019; 10:1835. 10.1038/s41467-019-09735-4 31015435 PMC6478834

[67] Li L, Zou J, Zhou M, Li H, Zhou T, Liu X, Huang Q, Yang S, Xiang Q, Yu R. Phenylsulfate-induced oxidative stress and mitochondrial dysfunction in podocytes are ameliorated by Astragaloside IV activation of the SIRT1/PGC1α /Nrf1 signaling pathway. Biomed Pharmacother. 2024; 177:117008. 10.1016/j.biopha.2024.117008 38901196

[68] Peng H, Liu X, Ieong CA, Tou T, Tsai T, Zhu H, Liu Z, Liu P. A Metabolomics study of metabolites associated with the glomerular filtration rate. BMC Nephrol. 2023; 24:105. 10.1186/s12882-023-03147-9 37085754 PMC10122376

[69] Hamer HM, Jonkers D, Venema K, Vanhoutvin S, Troost FJ, Brummer RJ. Review article: the role of butyrate on colonic function. Aliment Pharmacol Ther. 2008; 27:104–19. 10.1111/j.1365-2036.2007.03562.x 17973645

[70] Morrison DJ, Preston T. Formation of short chain fatty acids by the gut microbiota and their impact on human metabolism. Gut Microbes. 2016; 7:189–200. 10.1080/19490976.2015.1134082 26963409 PMC4939913

[71] Huda MN, Kim M, Bennett BJ. Modulating the Microbiota as a Therapeutic Intervention for Type 2 Diabetes. Front Endocrinol (Lausanne). 2021; 12:632335. 10.3389/fendo.2021.632335 33897618 PMC8060771

[72] Gaike AH, Paul D, Bhute S, Dhotre DP, Pande P, Upadhyaya S, Reddy Y, Sampath R, Ghosh D, Chandraprabha D, Acharya J, Banerjee G, Sinkar VP, et al. The Gut Microbial Diversity of Newly Diagnosed Diabetics but Not of Prediabetics Is Significantly Different from That of Healthy Nondiabetics. mSystems. 2020; 5:e00578-19. 10.1128/mSystems.00578-19 32234773 PMC7112960

[73] Wang Y, Ye X, Ding D, Lu Y. Characteristics of the intestinal flora in patients with peripheral neuropathy associated with type 2 diabetes. J Int Med Res. 2020; 48:300060520936806. 10.1177/0300060520936806 32938282 PMC7503028

[74] Wu C, Borné Y, Gao R, López Rodriguez M, Roell WC, Wilson JM, Regmi A, Luan C, Aly DM, Peter A, Machann J, Staiger H, Fritsche A, et al. Elevated circulating follistatin associates with an increased risk of type 2 diabetes. Nat Commun. 2021; 12:6486. 10.1038/s41467-021-26536-w 34759311 PMC8580990

[75] Pan J, Nilsson J, Engström G, De Marinis Y. Elevated circulating follistatin associates with increased risk of mortality and cardiometabolic disorders. Nutr Metab Cardiovasc Dis. 2024; 34:418–25. 10.1016/j.numecd.2023.09.012 38000997

[76] Gaeini Z, Bahadoran Z, Mirmiran P. Saturated Fatty Acid Intake and Risk of Type 2 Diabetes: An Updated Systematic Review and Dose-Response Meta-Analysis of Cohort Studies. Adv Nutr. 2022; 13:2125–35. 10.1093/advances/nmac071 36056919 PMC9776642

[77] Wu T, Xu F, Su C, Li H, Lv N, Liu Y, Gao Y, Lan Y, Li J. Alterations in the Gut Microbiome and Cecal Metabolome During *Klebsiella pneumoniae*-Induced Pneumosepsis. Front Immunol. 2020; 11:1331. 10.3389/fimmu.2020.01331 32849494 PMC7411141

[78] Tucker JD, Doddapaneni R, Lu PJ, Lu QL. Ribitol alters multiple metabolic pathways of central carbon metabolism with enhanced glycolysis: A metabolomics and transcriptomics profiling of breast cancer. PLoS One. 2022; 17:e0278711. 10.1371/journal.pone.0278711 36477459 PMC9728907

[79] Shinohara M, Tashiro Y, Suzuki K, Fukumori A, Bu G, Sato N. Interaction between *APOE* genotype and diabetes in cognitive decline. Alzheimers Dement (Amst). 2020; 12:e12006. 10.1002/dad2.12006 32211501 PMC7085280

[80] Ganguli M, Beer JC, Zmuda JM, Ryan CM, Sullivan KJ, Chang CH, Rao RH. Aging, Diabetes, Obesity, and Cognitive Decline: A Population-Based Study. J Am Geriatr Soc. 2020; 68:991–8. 10.1111/jgs.16321 32020605 PMC8597580

[81] Monickaraj F, Aravind S, Gokulakrishnan K, Sathishkumar C, Prabu P, Prabu D, Mohan V, Balasubramanyam M. Accelerated aging as evidenced by increased telomere shortening and mitochondrial DNA depletion in patients with type 2 diabetes. Mol Cell Biochem. 2012; 365:343–50. 10.1007/s11010-012-1276-0 22411737

[82] Mahley RW, Rall SC Jr. Apolipoprotein E: far more than a lipid transport protein. Annu Rev Genomics Hum Genet. 2000; 1:507–37. 10.1146/annurev.genom.1.1.507 11701639

[83] Seshasai RK, Katz R, de Boer IH, Siscovick D, Shlipak MG, Rifkin DE, Sarnak MJ. Apolipoprotein E and kidney function in older adults. Clin Nephrol. 2012; 78:174–80. 10.5414/cn107427 22874105 PMC3874583

[84] Corbo RM, Scacchi R, Cresta M. Differential reproductive efficiency associated with common apolipoprotein e alleles in postreproductive-aged subjects. Fertil Steril. 2004; 81:104–7. 10.1016/j.fertnstert.2003.05.029 14711551

[85] Abdullah L, Evans JE, Emmerich T, Crynen G, Shackleton B, Keegan AP, Luis C, Tai L, LaDu MJ, Mullan M, Crawford F, Bachmeier C. APOE ε4 specific imbalance of arachidonic acid and docosahexaenoic acid in serum phospholipids identifies individuals with preclinical Mild Cognitive Impairment/Alzheimer's Disease. Aging (Albany NY). 2017; 9:964–85. 10.18632/aging.101203 28333036 PMC5391242

[86] Wang Y, Liu H, Ye Y, Fang W, Lin A, Dai X, Ye Q, Chen X, Zhang J. ApoE2 affects insulin signaling in the hippocampus and spatial cognition of aged mice in a sex-dependent manner. Cell Commun Signal. 2025; 23:112. 10.1186/s12964-025-02093-3 40011916 PMC11866816

[87] Dunk MM, Driscoll I, and Alzheimer’s Disease Neuroimaging Initiative. Total Cholesterol and APOE-Related Risk for Alzheimer's Disease in the Alzheimer's Disease Neuroimaging Initiative. J Alzheimers Dis. 2022; 85:1519–28. 10.3233/JAD-215091 34958023 PMC10442640

[88] Shinohara M, Kanekiyo T, Yang L, Linthicum D, Shinohara M, Fu Y, Price L, Frisch-Daiello JL, Han X, Fryer JD, Bu G. APOE2 eases cognitive decline during Aging: Clinical and preclinical evaluations. Ann Neurol. 2016; 79:758–74. 10.1002/ana.24628 26933942 PMC5010530

[89] Toledo JB, Arnold M, Kastenmüller G, Chang R, Baillie RA, Han X, Thambisetty M, Tenenbaum JD, Suhre K, Thompson JW, John-Williams LS, MahmoudianDehkordi S, Rotroff DM, et al, and Alzheimer's Disease Neuroimaging Initiative and the Alzheimer Disease Metabolomics Consortium. Metabolic network failures in Alzheimer's disease: A biochemical road map. Alzheimers Dement. 2017; 13:965–84. 10.1016/j.jalz.2017.01.020 28341160 PMC5866045

[90] He X, Huang Y, Li B, Gong CX, Schuchman EH. Deregulation of sphingolipid metabolism in Alzheimer's disease. Neurobiol Aging. 2010; 31:398–408. 10.1016/j.neurobiolaging.2008.05.010 18547682 PMC2829762

[91] Yin F. Lipid metabolism and Alzheimer's disease: clinical evidence, mechanistic link and therapeutic promise. FEBS J. 2023; 290:1420–53. 10.1111/febs.16344 34997690 PMC9259766

[92] Lefterov I, Wolfe CM, Fitz NF, Nam KN, Letronne F, Biedrzycki RJ, Kofler J, Han X, Wang J, Schug J, Koldamova R. APOE2 orchestrated differences in transcriptomic and lipidomic profiles of postmortem AD brain. Alzheimers Res Ther. 2019; 11:113. 10.1186/s13195-019-0558-0 31888770 PMC6937981

[93] Heffernan AL, Chidgey C, Peng P, Masters CL, Roberts BR. The Neurobiology and Age-Related Prevalence of the ε4 Allele of Apolipoprotein E in Alzheimer's Disease Cohorts. J Mol Neurosci. 2016; 60:316–24. 10.1007/s12031-016-0804-x 27498201 PMC5531868

[94] Beker N, Sikkes SAM, Hulsman M, Tesi N, van der Lee SJ, Scheltens P, Holstege H. Longitudinal Maintenance of Cognitive Health in Centenarians in the 100-plus Study. JAMA Netw Open. 2020; 3:e200094. 10.1001/jamanetworkopen.2020.0094 32101309 PMC7137688

[95] Corrada MM, Paganini-Hill A, Berlau DJ, Kawas CH. Apolipoprotein E genotype, dementia, and mortality in the oldest old: the 90+ Study. Alzheimers Dement. 2013; 9:12–8. 10.1016/j.jalz.2011.12.004 23123227 PMC3543489

[96] Kaup AR, Nettiksimmons J, Harris TB, Sink KM, Satterfield S, Metti AL, Ayonayon HN, Yaffe K, and Health, Aging, and Body Composition (Health ABC) Study. Cognitive resilience to apolipoprotein E ε4: contributing factors in black and white older adults. JAMA Neurol. 2015; 72:340–8. 10.1001/jamaneurol.2014.3978 25599330 PMC4624320

[97] Wilmanski T, Rappaport N, Earls JC, Magis AT, Manor O, Lovejoy J, Omenn GS, Hood L, Gibbons SM, Price ND. Blood metabolome predicts gut microbiome α-diversity in humans. Nat Biotechnol. 2019; 37:1217–28. 10.1038/s41587-019-0233-9 31477923

[98] Salek RM, Steinbeck C, Viant MR, Goodacre R, Dunn WB. The role of reporting standards for metabolite annotation and identification in metabolomic studies. Gigascience. 2013; 2:13. 10.1186/2047-217X-2-13 24131531 PMC3853013

[99] Kokla M, Virtanen J, Kolehmainen M, Paananen J, Hanhineva K. Random forest-based imputation outperforms other methods for imputing LC-MS metabolomics data: a comparative study. BMC Bioinformatics. 2019; 20:492. 10.1186/s12859-019-3110-0 31601178 PMC6788053

[100] Benjamini Y, Hochberg Y. Controlling the False Discovery Rate: A Practical and Powerful Approach to Multiple Testing. J R Stat Soc Ser B Methodol. 1995; 57:289–300. 10.1111/j.2517-6161.1995.tb02031.x

