# Supplementary material for: APOE genotype and biological age impact inter-omic associations related to bioenergetics
Source: Aging (Albany NY). 2025 May 3;17(5):1105–38. doi: 10.18632/aging.206243 (PMC12151507; doi:10.18632/aging.206243)
Supplement: Supplementary Figures [file aging-17-206243-s001.pdf]

## SUPPLEMENTARY FIGURES

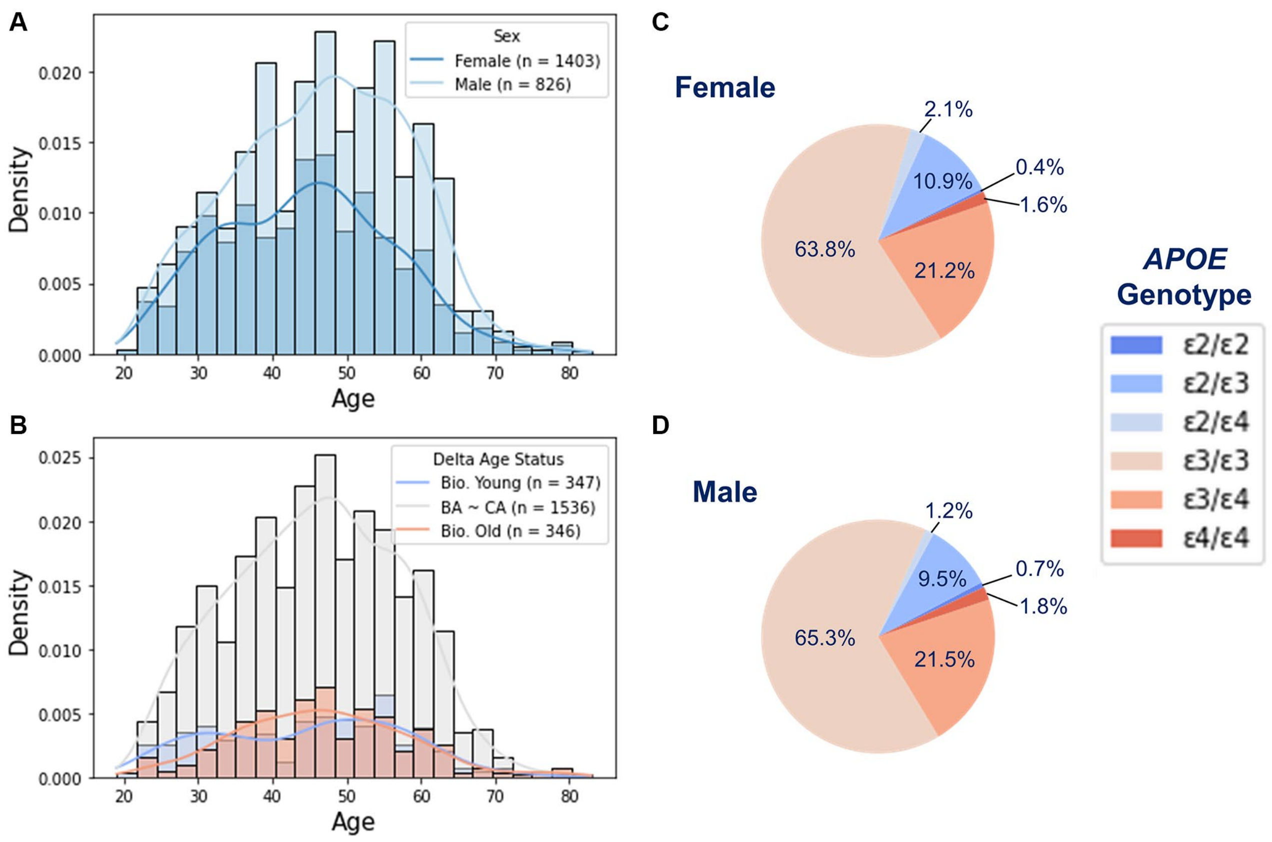


**Supplementary Figure 1. The Arivale cohort contains a range of community dwelling individuals spread across ages, delta age statuses, and *APOE* genotypes.** (**A**, **B**) Density histograms of baseline chronological ages in the Arivale cohort stratified by sex (**A**) and delta age status, with biologically young and old defined as having a biological age 7.5 years younger or older than chronological age, respectively (**B**). The lines indicate the kernel density estimates. (**C**, **D**) Pie charts displaying *APOE* genotype frequencies in the female (**C**) and male (**D**) Arivale participants. Presented is the baseline data used in interaction analyses.

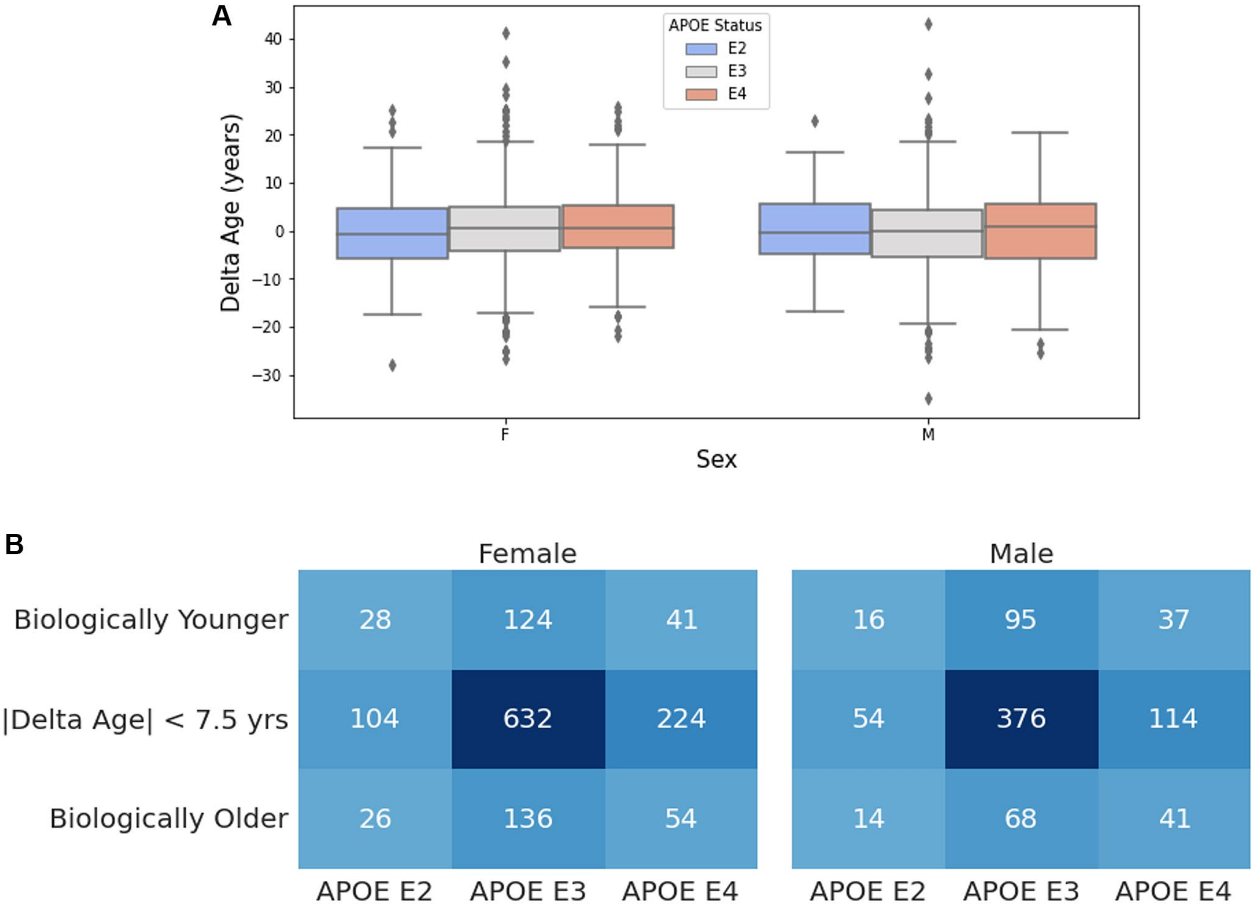


**Supplementary Figure 2. Delta age and delta age-based stratifications are not significantly different across APOE statuses for either males or females in Arivale.** (**A**) Box plot of delta age across APOE statuses. Pairwise Mann–Whitney *U*-tests between APOE statuses within male and female showed non-significant *p*-values (smallest *p*-value = 0.062 between female E2 and E4). *n* = 158 (Female E2), *n* = 892 (Female E3), *n* = 319 (Female E4), *n* = 84 (Male E2), *n* = 539 (Male E3), *n* = 192 (Male E4). (**B**) Counts of individuals in APOE and delta age categories, stratified by sex. The chi-squared tests yielded *p* = 0.59 for females and *p* = 0.041 for males.

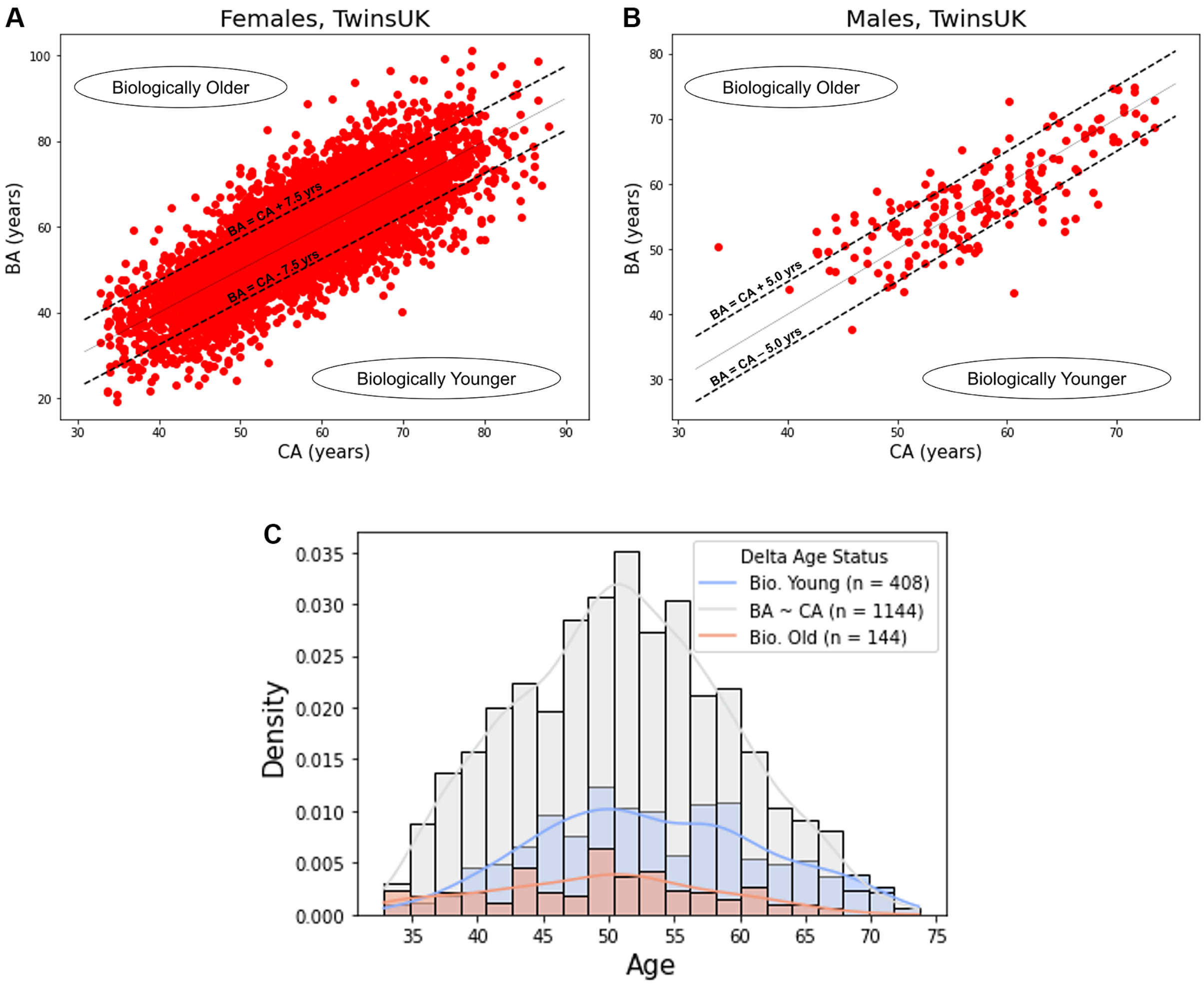


**Supplementary Figure 3. Metabolomic BA was predicted by fitting a model to TwinsUK data.** (**A**, **B**) The scatterplot of BA and CA for female (*n* = 1,635 individuals, Pearson's r = 0.778) (A) and male (*n* = 61, r = 0.776) (**B**) TwinsUK participants. The solid line indicates BA = CA, and the dotted lines indicate cutoffs for defining the Biologically Younger and Older groups. See Methods for model details. (**C**) A density histogram of baseline chronological ages in the TwinsUK cohort stratified by delta age status. The lines indicate the kernel density estimates.

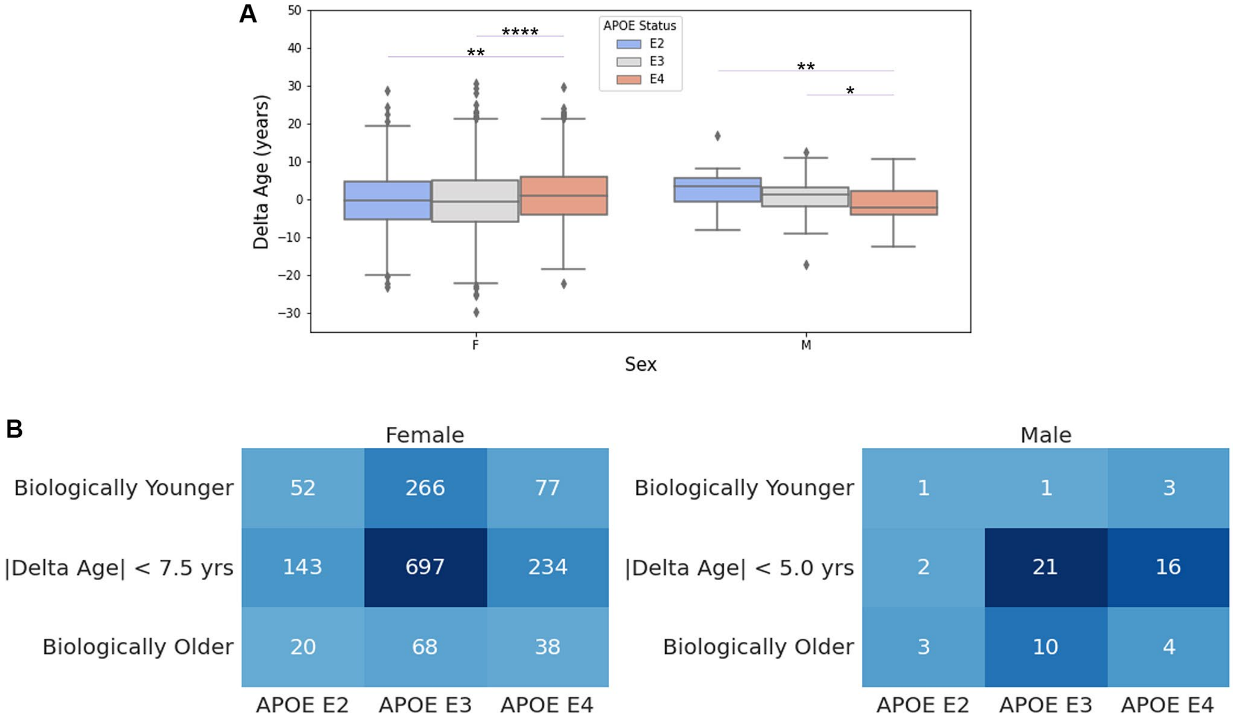


**Supplementary Figure 4. Delta age is significantly different across APOE statuses in the TwinsUK cohort.** (**A**) Box plot of delta age across APOE statuses for all TwinsUK participants, including longitudinal. $^{*}p < 0.05$, $^{**}p < 0.01$, $^{***}p < 0.001$, $^{****}p < 0.00001$ based on pairwise Mann-Whitney *U*-tests. $n$ = 641 (Female E2), $n$ = 3050 (Female E3), $n$ = 1043 (Female E4), $n$ = 18 (Male E2), $n$ = 95 (Male E3), $n$ = 69 (Male E4). (**B**) Counts of individuals in APOE and delta age categories at baseline visit, stratified by sex. The chi-squared tests yielded $p$ = 0.26 for females and $p$ = 0.08 for males.

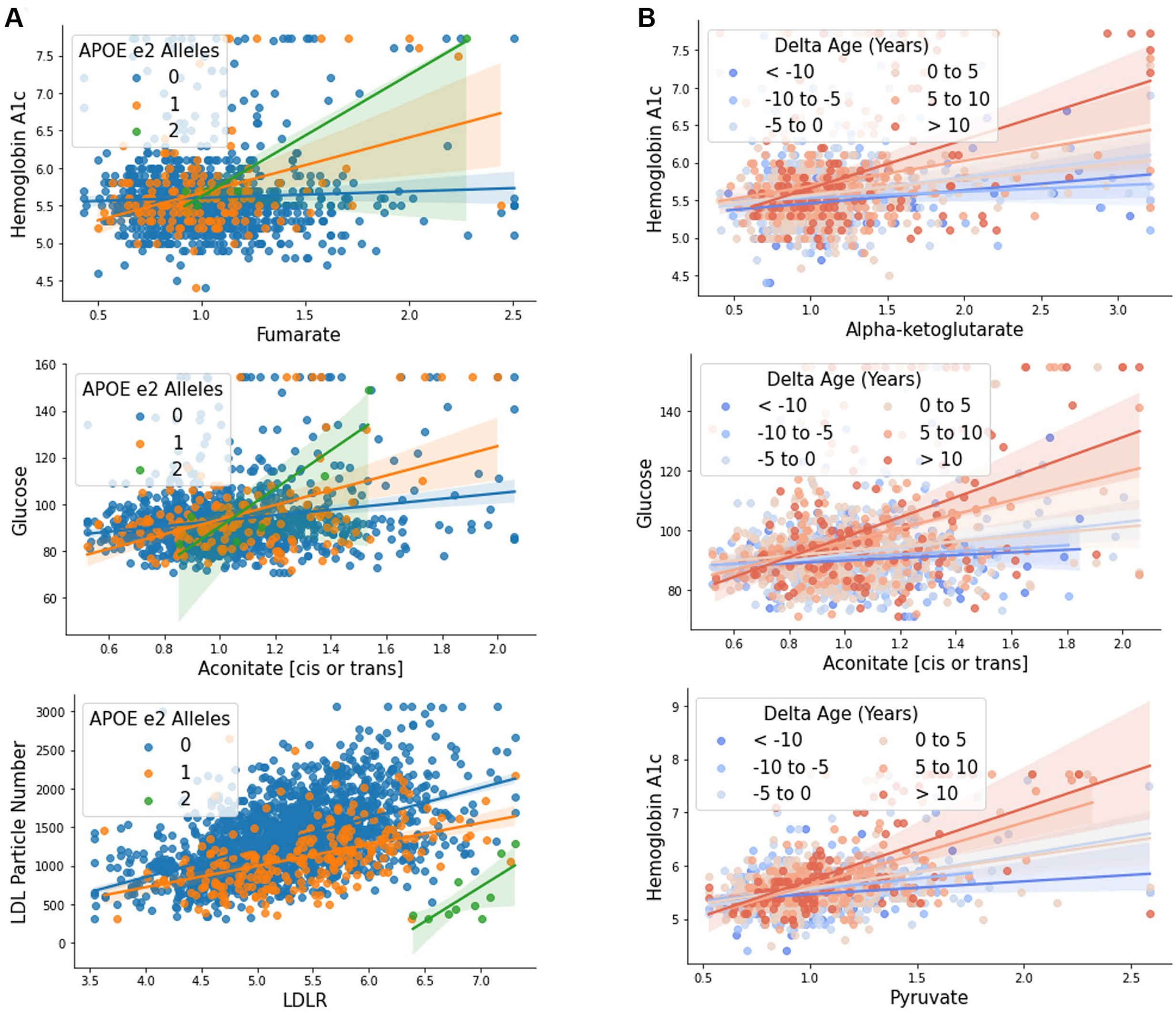


**Supplementary Figure 5. Inter-omic associations are modified by ε2 allele dosage and continuous delta age.** Scatter plots of inter-omic analyte pairs with associations significantly modified by *APOE* ε2 allele dosage (**A**) and by delta age (**B**). Line indicates simple linear regression, with shading indicating the 95% confidence interval.

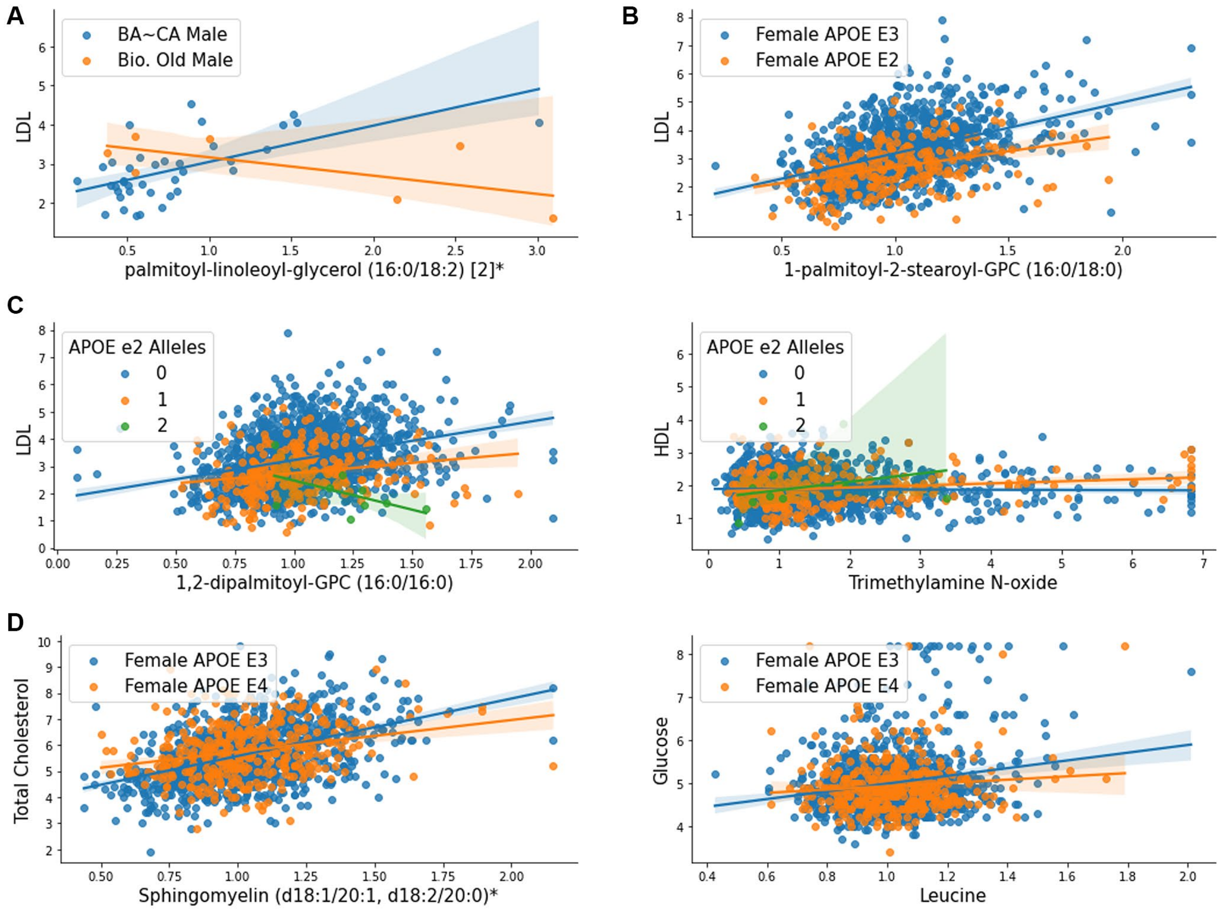


**Supplementary Figure 6. Inter-omic associations involving lipids are modified by APOE and delta age statuses in the TwinsUK validation cohort.** Scatter plots of inter-omic analyte pairs with associations significantly modified by biological oldness in males (**A**), APOE E2 in females (**B**), the APOE ε2 allele (**C**), APOE E4 in females (**D**) in the TwinsUK cohort. Line indicates simple linear regression, with shading indicating the 95% confidence interval.
