# Supplementary material for: APOE genotype and biological age impact inter-omic associations related to bioenergetics
Source: Aging (Albany NY). 2025 May 3;17(5):1105–38. doi: 10.18632/aging.206243 (PMC12151507; doi:10.18632/aging.206243)
Supplement: Supplementary Tables [file aging-17-206243-s002.pdf]

SUPPLEMENTARY TABLES

Supplementary Table 1. Enriched metabolic sub-pathways in the metabolites significantly associated with APOE or delta age statuses.

	Enriched in positive associations		Enriched in negative associations	
Arivale	*Sub-Pathway*	*pFDR*	*Sub-Pathway*	*pFDR*
**APOE E2**	**Diacylglycerol**	**2.6e-12**	Sphingolipid Metabolism	0.874
	**Steroid**	**0.088**	**Plasmalogen**	**4.3e-5**
**Biologically young**	Endocannabinoid	0.255	Dipeptide	0.319
	Phosphatidylserine (PS)	0.933	Ceramide PEs	0.816
	**Plasmalogen**	**8.5e-8**	Steroid	1.000
**Biologically old**	**Polyamine Metabolism**	**0.030**		
	Histidine Metabolism	0.428		

Presented are the metabolite sub-pathways, as categorized by the Metabolon platform, enriched with $p < 0.05$ in the metabolites that exhibited significantly positive or negative associations with APOE or delta age statuses after FDR correction (pFDR < 0.1). Bolding denotes pFDR < 0.1 (Benjamini–Hochberg method) of the enrichment analysis.

Supplementary Table 2. Enriched metabolic sub-pathways in the metabolites associated pre-adjustment with APOE or delta age statuses.

	Enriched in positive associations		Enriched in negative associations	
Arivale	*Sub-Pathway*	*p-value*	*Sub-Pathway*	*p-value*
	**Diacylglycerol**	**2.70E-16**	**Sphingolipid Metabolism**	**2.70E-07**
**APOE E2**	**Plasmalogen**	**0.002**	Sphingomyelins	**0.026**
	**Long Chain Fatty Acid**	**0.002**	Ceramide PEs	**0.026**
	Monoacylglycerol	0.027		
	**Lysolipid**	**0.001**	Plasmalogen	0.005
	Diacylglycerol	0.003	Androgenic Steroids	0.035
**APOE E4**	Monoacylglycerol	0.013	Methionine, Cysteine, SAM and Taurine Metabolism	0.038
	Polyamine Metabolism	0.043		
	**Long Chain Fatty Acid**	**2.30E-05**	**Plasmalogen**	**5.50E-07**
	Steroid	0.007	Urea cycle; Arginine and Proline Metabolism	0.011
	Fatty Acid Metabolism (Acyl Carnitine)	0.019	Leucine, Isoleucine and Valine Metabolism	
**Biologically young**	Endocannabinoid		Carnitine Metabolism	0.013
		0.026	Fatty Acid Metabolism (Acyl Choline)	
			Histidine Metabolism	0.014
				0.04
				0.046
	**Plasmalogen**	**6.60E-09**	Steroid	0.021
	**Leucine, Isoleucine and Valine Metabolism**	**1.60E-04**	Tryptophan Metabolism	0.022
	**Polyamine Metabolism**			
**Biologically old**	Urea cycle; Arginine and Proline Metabolism	**0.001**		
	Histidine Metabolism	0.017		
	Phospholipid Metabolism			
		0.027		
		0.048		

TwinsUK Validation				
**APOE E2**	**Diacylglycerol**	**1.50E-05**	**Sphingolipid Metabolism**	**4.00E-06**
	Steroid	0.01		
**APOE E4**	**Lysolipid**	**2.20E-11**	**Xanthine Metabolism**	**1.90E-04**
	**Monoacylglycerol**	**3.00E-06**	Ketone Bodies	0.029
	**Diacylglycerol**	**0.001**		

Presented are the metabolite sub-pathways, as categorized by the Metabolon platform, enriched with $p < 0.05$ in the metabolites that exhibited positive or negative associations with APOE or delta age statuses with pre-adjusted $p < 0.05$. Bolding denotes pFDR < 0.1 (Benjamini–Hochberg method) of the enrichment analysis.

Supplementary Table 3. Metabolites significantly associated with delta age and APOE statuses in stratified chronological age tertiles.

Arivale CA Tertile	Bottom (18–43) Years		Middle (43–53) Years		Top (53–87) Years	
	Metabolite	***β***	***Metabolite***	***β***	***Metabolite***	***β***
Biologically Young	sphingosine	0.582				
Biologically Old	1,5-anhydroglucitol (1,5-AG)	−0.498	N2,N5-diacetylornithine	0.152	3-hydroxybutyrate (BHBA)	0.465
	X - 11372	−0.470	urea	0.538	(R)-3-hydroxybutyrylcarnitine	0.441
	X - 11880	−0.449	1-arachidonoyl-GPE (20:4n6)*	0.515	docosadioate (C22-DC)	−0.404
	X - 11378	−0.451	tiglylcarnitine (C5:1-DC)	0.523	histidine	−0.376
	N-palmitoyl-sphinganine (d18:0/16:0)	0.466	arabitol/xylitol	0.467	5-oxoproline	−0.348
	1-(1-enyl-stearoyl)-2-linoleoyl-GPE (P-18:0/18:2)*	0.436	isovalerylglycine	0.506	linoleoyl-linolenoyl-glycerol (18:2/18:3) [2]*	−0.398
	3beta-hydroxy-5-cholestenoate	−0.394	1-oleoyl-GPE (18:1)	0.463	hexanoylglutamine	0.393
	X - 16935	−0.362	N-acetylvaline	0.506	branched-chain, straight-chain, or cyclopropyl 10:1 fatty acid (1)*	0.378
	linolenate (alpha or gamma; (18:3n3 or 6))	−0.409	isobutyrylglycine	0.471	6-bromotryptophan	−0.393
	glycosyl-N-stearoyl-sphingosine (d18:1/18:0)	0.402	1-methylhistidine	0.451	1-pentadecanoyl-GPC (15:0)*	−0.355
APOE E2			androsterone sulfate	0.488	oleoyl-arachidonoyl-glycerol (18:1/20:4) [2]*	0.453
			5alpha-androstan-3alpha,17beta-diol monosulfate (1)	0.481	stearoyl-arachidonoyl-glycerol (18:0/20:4) [2]*	0.454
					stearoyl-arachidonoyl-glycerol (18:0/20:4) [1]*	0.451
APOE E4	linoleoyl-arachidonoyl-glycerol (18:2/20:4) [1]*	0.357				
	palmitoleoyl-arachidonoyl-glycerol (16:1/20:4) [2]*	0.346				
**TwinsUK CA Tertile**	**Bottom, (32.85–47.72) Years**		**Middle, (47.72–55.05) Years**		**Top, (55.05–73.69) Years**	
TwinsUK	*Metabolite*	*β*	*Metabolite*	*β*	*Metabolite*	*β*
APOE E2					X - 21736	0.466

					butyrylcarnitine (C4)	0.414
APOE E4	1-palmitoylglycerol (16:0)	0.413			X - 24065	0.401
	2-oleoylglycerol (18:1)	0.370			N-acetylcitrulline	−0.379
	2-palmitoylglycerol (16:0)	0.373			1-oleoylglycerol (18:1)	0.348
	1-dihomo-linolenylglycerol (20:3)	0.366				
	1-myristoylglycerol (14:0)	0.317				

Metabolites with pFDR < 0.1 association in metabolite abundance GLMs in Arivale when stratified by chronological age (CA) tertiles are reported with their β-coefficient estimates. Only the 10 metabolites with the lowest *p*-value for the association with the biologically old group are shown out of 16, 98, and 31 with pFDR < 0.1 in the bottom, middle, and top CA tertiles (Supplementary File 2 for full data). Metabolite names ending in "*" indicate compounds not confirmed based on a standard but having high confidence in its identity.

Supplementary Table 4. Top ten inter-omic analyte pair associations modified by *APOE* allele dosage and delta age in Arivale.

***APOE* ε2 allele dosage**			***APOE* ε4 allele dosage**			**Delta age**		
++	X - 11372	Rikenellaceae_RC9_gut_group	−−	isoursodeoxycholate	Rikenellaceae_RC9_gut_group	++	Hemoglobin A1C	pyruvate
−−	N-acetylglutamate	Faecalibacterium	++	1-arachidonoyl-GPE (20:4n6)*	Tyzzerella	++	Hemoglobin A1C	mannose
−−	LDL particle number	LDLR	−−	1-arachidonoyl-GPI (20:4)*	Dorea	++	Glucose	pyruvate
−−	LDL small particle number	LDLR	−−	isoursodeoxycholate	Prevotellaceae_UCG-001	++	Glucose	mannose
++	BMP6	N-palmitoylglycine	++	LDL Size	1-(1-enyl-palmitoyl)-2-oleoyl-GPC (P-16:0/18:1)*	++	Glucose	gluconate
++	Hemoglobin A1C	fumarate	−	isoursodeoxycholate	Prevotellaceae_Ga6A1_group	++	Glucose	CD163
−−	4-hydroxychlorothalonil	Anaerotruncus	+	IL17C	12,13-DiHOME	++	Hemoglobin A1C	2-hydroxybutyrate/2-hydroxyisobutyrate
++	Potassium	2-aminoheptanoate	−	KITLG	glucose	++	Glucose	fructose
++	Glucose	3-hydroxy-2-ethylpropionate	+	stachydrine	Romboutsia	++	Hemoglobin A1C	SELE
−−	4-hydroxychlorothalonil	DTU089	−	KITLG	glucose	−−	Glucose	KITLG

For each set of models, the ten analyte pairs with the lowest *p*-values for the interaction term representing the modification of *APOE* allele dosage or delta age on the association between the two analytes are tabulated. '+' and '−' indicate positive and negative interaction terms, respectively, with '++' and '−−' indicating pFDR > 0.1 (Supplementary File 3 for full data). Underlining indicates a metabolite associated with the experimental group in the analysis of differential metabolite abundance (with pre-adjusted *p* < 0.05). Metabolite names ending in "*" indicate compounds not confirmed based on a standard but having high confidence in its identity.
